# Global incidence of esophageal cancer by histological and anatomical subtypes

**DOI:** 10.1038/s41467-026-76540-1

**Published:** 2026-09-01

**Authors:** Claire Chenwen Zhong, Junjie Huang, Zehuan Yang, Zhaojun Li, Lin Zhang, Xu Lin, Don Eliseo Lucero-Prisno, Wanghong Xu, Minmin Wang, Yinzi Jin, Zhi-Jie Zheng, Mellissa Withers, Martin CS Wong

**Affiliations:** 1 The Jockey Club School of Public Health and Primary Care, Faculty of Medicine, Chinese University of Hong Kong, Hong Kong SAR, China; 2 Centre for Health Education and Health Promotion, Faculty of Medicine, The Chinese University of Hong Kong, Hong Kong SAR, China; 3 The School of Public Health and Preventive Medicine, Monash University, Melbourne, VIC Australia; 4 Department of Thoracic Surgery, The First Affiliated Hospital, School of Medicine, Zhejiang University, Hangzhou, Zhejiang China; 5 Department of Global Health and Development, London School of Hygiene and Tropical Medicine, London, United Kingdom; 6 School of Public Health, Fudan University, Shanghai, China; 7 Department of Global Health, School of Public Health, Peking University, Beijing, China; 8 Department of Population and Health Sciences, Institute for Global Health, University of Southern California, Los Angeles, CA USA

**Keywords:** Risk factors, Public health

## Abstract

Esophageal cancer is a major contributor to the global cancer burden, yet comprehensive global assessments of its burden across subtypes are lacking. This study examines the incidence, risk factors, and trends of esophageal cancer by histological and anatomical subtypes, with stratification by sex and age across different countries, using GLOBOCAN 2022 estimates, Cancer Incidence in Five Continents data, and country-level socioeconomic, lifestyle, and metabolic risk-factor indicators. Here we show that Eastern Asia has the highest age-standardized rate (ASR) of incidence of esophageal squamous cell carcinoma (ESCC) (7.2 per 100,000 population) and upper- and middle-third esophageal cancer (6.0), whereas Northern Europe exhibits the highest ASR of incidence of esophageal adenocarcinoma (EAC) (3.2) and lower‑third esophageal cancer (3.6). EAC and lower‑third esophageal cancer generally share risk factors and exhibit increasing trends; in contrast, ESCC and the upper- and middle-third esophageal cancer share a different risk profile and show an overall decreasing trend. In developed regions, EAC and lower‑third esophageal cancer are more common, whereas in developing regions, ESCC and upper- and middle-third esophageal cancer predominate. Our findings may assist the development of tailored prevention strategies based on locally emerging patterns of esophageal cancer subtypes and relevant risk factors.

## Abstract (web summary)

There is a need for more insights into subtype differences, temporal trends and related risk factors of oesophageal cancer. Here, the authors find that the incidence, risk factors, and trends of oesophageal cancer vary significantly by histological and anatomical subtypes in different populations globally.

## Introduction

Esophageal cancer is a common malignancy, with more than 600,000 new cases reported worldwide in 2020, accounting for ~3% of all cancers; in the same year, 544,076 deaths were attributed to the disease^1^. Esophageal cancer has two main histological subtypes: squamous cell carcinoma (ESCC) and adenocarcinoma (EAC); and three anatomical subtypes: upper-, middle- (UpMid) and lower-third (Low) esophageal cancer^2,3^. ESCC and EAC are distinctively different from each other in terms of epidemiology, risk factors and prognosis^2–4^. Despite substantial improvements in esophageal cancer survival over recent decades^2,3,5–8^, survival gains have been less evident at three years post‑diagnosis and among older patients and those with ESCC^2^. These disparities highlight the need for ongoing surveillance of incidence and temporal trends by histological subtype, age, and sex to support evidence‑based policymaking and early diagnosis^2,9–13^.

Some risk factors of esophageal cancer have been identified. For instance, both histological subtypes are associated with smoking and low fruit and vegetable consumption, while obesity, Barrett’s esophagus, and gastroesophageal reflux disease are linked specifically to EAC, and alcohol consumption is linked specifically to ESCC^14^. Previous studies have explored the burden and risk factors for different subtypes of esophageal cancer, but important research gaps remain as they were limited to specific populations or countries^4,9^. Studies from the Global Burden of Disease (GBD), despite offering comprehensive global analyses, lack insights on subtype differences^10–13^. Moreover, available subtype-specific international and global analyses have either relied on earlier incidence data, focused mainly on histological subtypes, or did not jointly evaluate anatomical subtypes, temporal trends, and country-level risk-factor associations ^15–17^.

To address these gaps, this study provides a comprehensive and contemporary global assessment of esophageal cancer incidence, country-level risk-factor associations, and temporal trends by histological and anatomical subtypes and key population subgroups.

In this work, a substantial increase in the proportion and incidence of EAC/Low esophageal cancer is observed in selected populations. Given the positive associations of EAC/Low incidence with human development index (HDI) and the gross domestic product (GDP) per capita, these increasing trends are likely to persist with ongoing global economic development. ESCC and UpMid subtypes share similar risk factors, whereas EAC and Low subtypes exhibit a distinct risk‑factor profile, underscoring the need for prevention strategies tailored to specific histological and anatomical subtypes. Developed economies should prioritize the prevention and management of GERD and Barrett’s esophagus to reduce the burden of EAC/Low esophageal cancer, while developing regions may benefit from reducing tobacco and alcohol use and improving obesity management, given their strong influence on ESCC and UpMid subtypes. As ESCC and EAC differ in prognosis and survival, continued monitoring of subtype‑specific trends is essential to inform future policymaking and strategies for early detection. Further studies are warranted to continuously track global trends in esophageal cancer incidence and mortality by subtype.

## Results

### Esophageal cancer incidence by subtype in 2022

**Fig. 1:**
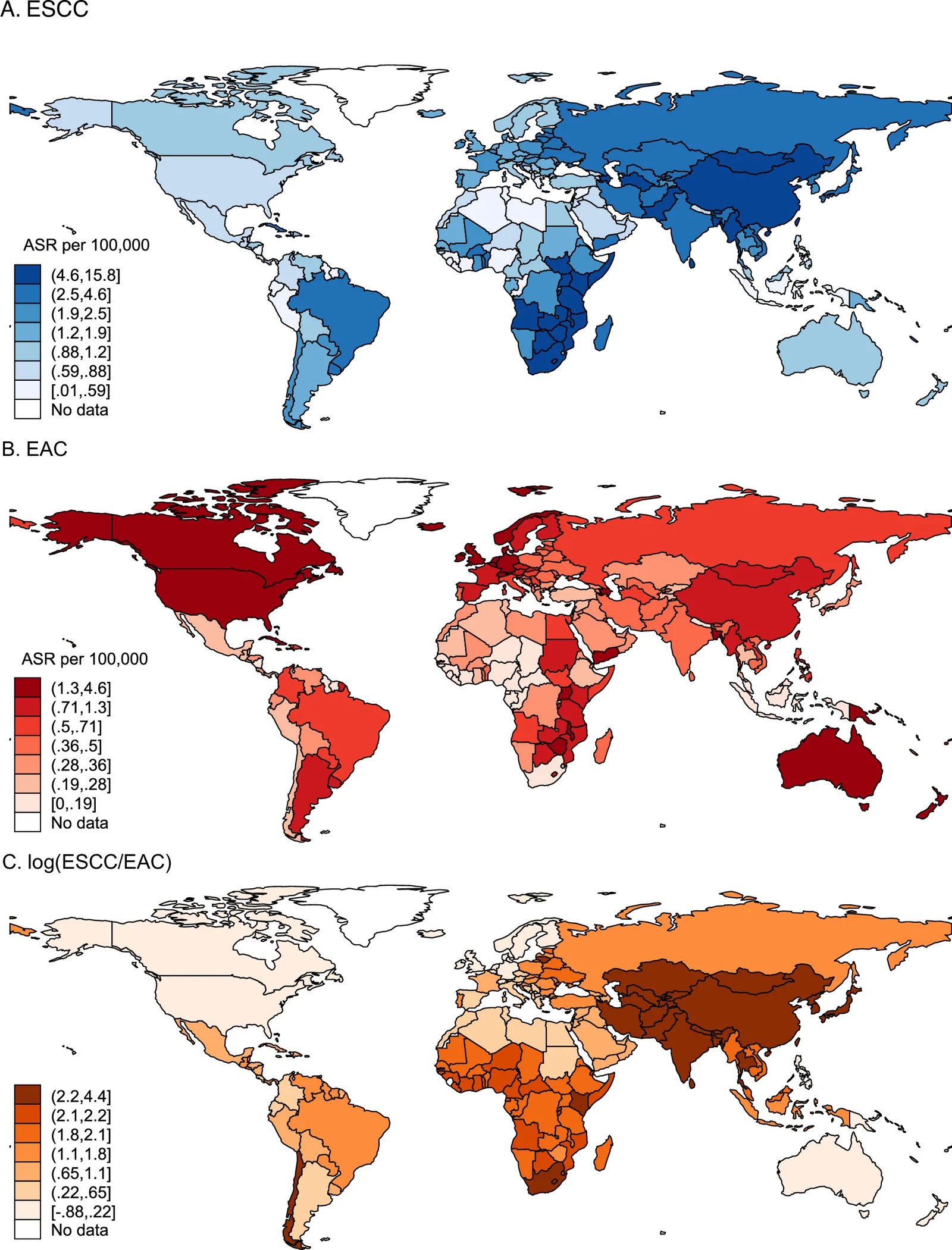
Global incidence of esophageal cancer by histological subtype. **A**–**C** show age‑standardized incidence rates in 2022 by country for ESCC, EAC, and the logarithm of the ESCC‑to‑EAC ratio. ASR age-standardized rate, ESCC esophageal squamous cell carcinoma, EAC esophageal adenocarcinoma.

**Fig. 2:**
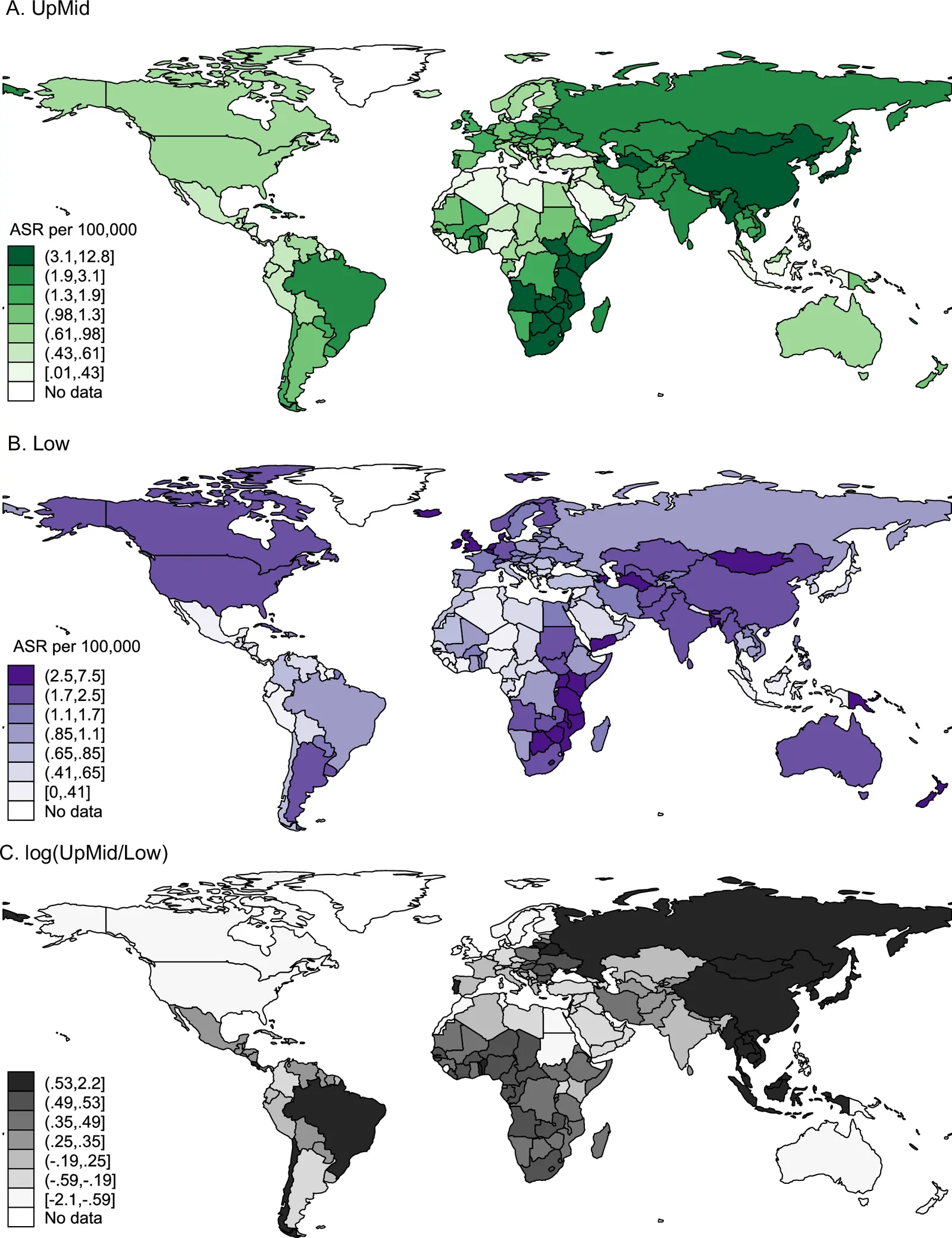
Global incidence of esophageal cancer by anatomical subtype. **A**–**C** show age‑standardized incidence rates in 2022 by country for UpMid, Low, and the logarithm of the UpMid‑to‑Low ratio. ASR age-standardized rate, UpMid upper- and middle-third esophageal cancer, Low lower-third esophageal cancer.

The total number of esophageal cancer cases was 510,910 across the world in 2022 (ASR: 5.0) (Supplementary Dataset 1). The highest and lowest ASR were observed in Eastern Asia (7.6) and Northern Africa (1.4), indicating a five-fold difference between regions. Malawi (17.9), Bangladesh (16.0) and Mongolia (15.7) reported the highest ASR. By histological subtype, the proportions of ESCC and EAC were 82.6% and 16.0%, respectively. The number of ESCC was 422,063 across the world (ASR: 4.1). The highest and lowest ASR of ESCC were observed in Eastern Asia (7.2) and Northern America (0.85), indicating an eight-fold difference between regions. Malawi (15.8), Bangladesh (14.6) and Mongolia (14.2) reported the highest ASR of ESCC (Fig. 1). The number of EAC cases was 81,827 across the world (ASR: 0.69). The highest and lowest ASR of EAC were observed in Northern Europe (3.2) and South-Eastern Asia (0.34), indicating a nine-fold difference between regions. The Netherlands (4.6), United Kingdom (3.9), and Ireland (2.9) reported the highest ASR of EAC among countries. By anatomical subtype, the proportions of esophageal cancer occurring in the upper and middle third (UpMid) and lower third (Low) were 63.4% and 36.6%. The number of UpMid cases was 323,788 across the world (ASR: 3.0). The highest and lowest ASR of UpMid were observed in Eastern Asia (6.0) and Northern Africa (0.59), indicating a tenfold difference between regions. Mongolia (12.8), Malawi (11.1), and Bangladesh (9.0) reported the highest ASR of UpMid among countries (Fig. 2). The number of Low cases was 187,122 across the world (ASR: 1.9). The highest and lowest ASR of Low were observed in Northern Europe (3.6) and Southern Europe (0.79), indicating a four-fold difference between regions. Uganda (7.5), Kenya (7.0), and Bangladesh (7.0) reported the highest ASR of Low among countries.

### Sex-specific esophageal cancer incidence by subtype in 2022

The esophageal cancer incidence in males (7.6, 365,126) was higher than in females (2.6, 145,784) (Supplementary Dataset 2). The highest ASR of esophageal cancer in males was found in Eastern Asia (12.2 vs. 3.4 in females), Northern Europe (7.6 vs. 2.6), and South-Central Asia (7.3 vs. 4.2). By histologic subtype, males had a higher ASR of ESCC (5.7 vs. 2.5 in females) and EAC (1.1 vs. 0.3). The proportion of ESCC was higher in females than males (86.0% in females vs 81.3% in males). By anatomical subtype, males had a higher ASR of UpMid (4.1 vs. 1.7 in females) and Low (2.7 vs. 1.1) compared with females. The proportion of UpMid was higher in females than males (66.4% in females vs 62.2% in males). In females, regions with the highest proportion of UpMid were Eastern Asia (79.3% in females vs. 73.6% in males), Southern Europe (65.6% in females vs. 55.3% in males) and Eastern Europe (65.4% in females vs. 64.2% in males).

### Age-specific esophageal cancer incidence by subtype in 2022

In the population aged 50–74, the ASR of esophageal cancer was 19.8 (334,899), which was higher than in the population aged 15–49 (1.1; 45,242) (Supplementary Dataset 3). The highest ASR of the population aged 50–74 was found in Eastern Asia (31.8 vs. 0.67 in the population aged 15–49), South-Central Asia (21.6 vs. 2.3), and Northern Europe (20.2 vs. 0.62). The population aged 15–49 had a slightly higher proportion of ESCC and lower proportion of EAC compared to the population aged 50–74 (ESCC: 87.3% in the population aged 15–49 vs. 83.8% in the population aged 50–74; EAC: 11.9% vs. 14.8%). As for the anatomical subtype, the population aged 50–74 had a higher incidence (UpMid: 12.7 vs. 0.71 in the population aged 15–49; Low: 7.2 vs. 0.52) but the population aged 15–49 had a higher proportion of Low than the population aged 50–74 (42.2% vs. 36.3%, respectively). The highest proportion of Low in the population aged 15–49 was found in Oceania (79.2% in the population aged 15–49 vs. 68.9%), Northern America (78.8% vs. 73.7%), and Caribbean (75.9% vs. 48.3%).

### Associations of risk factors with esophageal cancer incidence

At the country level, esophageal cancer incidence was positively associated with the prevalence of alcohol drinking (*β* = 0.016, 95% CI 0.001–0.030) and dietary risk factors (*β* = 0.035, 95% CI 0.015–0.056) but negatively associated with the prevalence of physical inactivity *(β* = −0.014, 95% CI −0.027 to −0.001), obesity (*β* = −0.018, 95% CI −0.030 to −0.007), and diabetes (*β* = −0.043, 95% CI −0.062 to −0.024). The ESCC/EAC esophageal cancer incidence ratio (ESCC/EAC) was positively associated with the prevalence of unhealthy diet (*β* = 0.037, 95% CI 0.016 to 0.059; Supplementary Dataset 4), but negatively associated with HDI (*β* = −0.338, 95% CI −0.417 to −0.258), GDP per capita (*β* = −0.232, 95% CI −0.290 to −0.174), and the prevalence of smoking (*β* = −0.055, 95% CI −0.072 to −0.038), alcohol drinking (*β* = −0.028, 95% CI −0.042 to −0.013), physical inactivity (*β* = −0.044, 95% CI −0.057 to −0.031), obesity (*β* = −0.047, 95% CI −0.057 to −0.036), diabetes (*β* = −0.057, 95% CI −0.076 to −0.037), and lipid disorder (*β* = −0.043, 95% CI −0.054 to −0.033) at a country level. Similar associations were also found for the UpMid/Low incidence ratio, except for non-significant association with alcohol drinking. The results of sensitivity analysis using 2012 data were consistent with the current findings (Supplementary Dataset 5).

### Associations of risk factors with esophageal cancer incidence by subtype

**Fig. 3:**
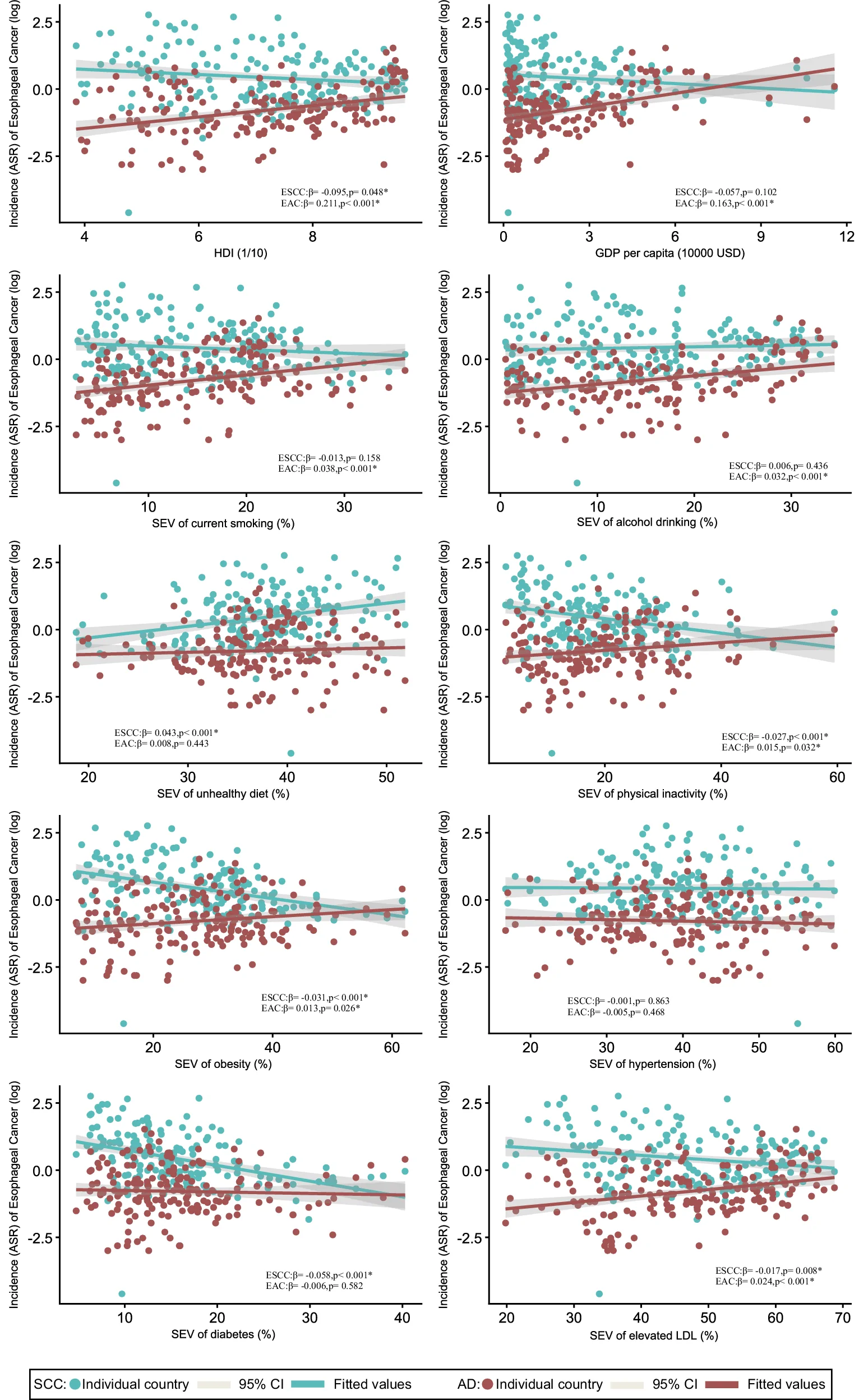
Associations between risk factors and esophageal cancer by histological subtype. Associations were evaluated using two-sided linear regression models with log-transformed age-standardized incidence rates as the outcome. Each dot represents an individual country (*n* = 185), and fitted regression lines with 95% confidence intervals are shown. β coefficients and exact P values are annotated in each panel, representing the change in log-transformed age-standardized incidence rate per unit increase in each risk-factor indicator. No adjustment was made for multiple comparisons. ASR age-standardized rate, ESCC esophageal squamous cell carcinoma, EAC esophageal adenocarcinoma, HDI Human Development Index, GDP Gross Domestic Product, SEV summary exposure value, LDL low-density lipoprotein.

**Fig. 4:**
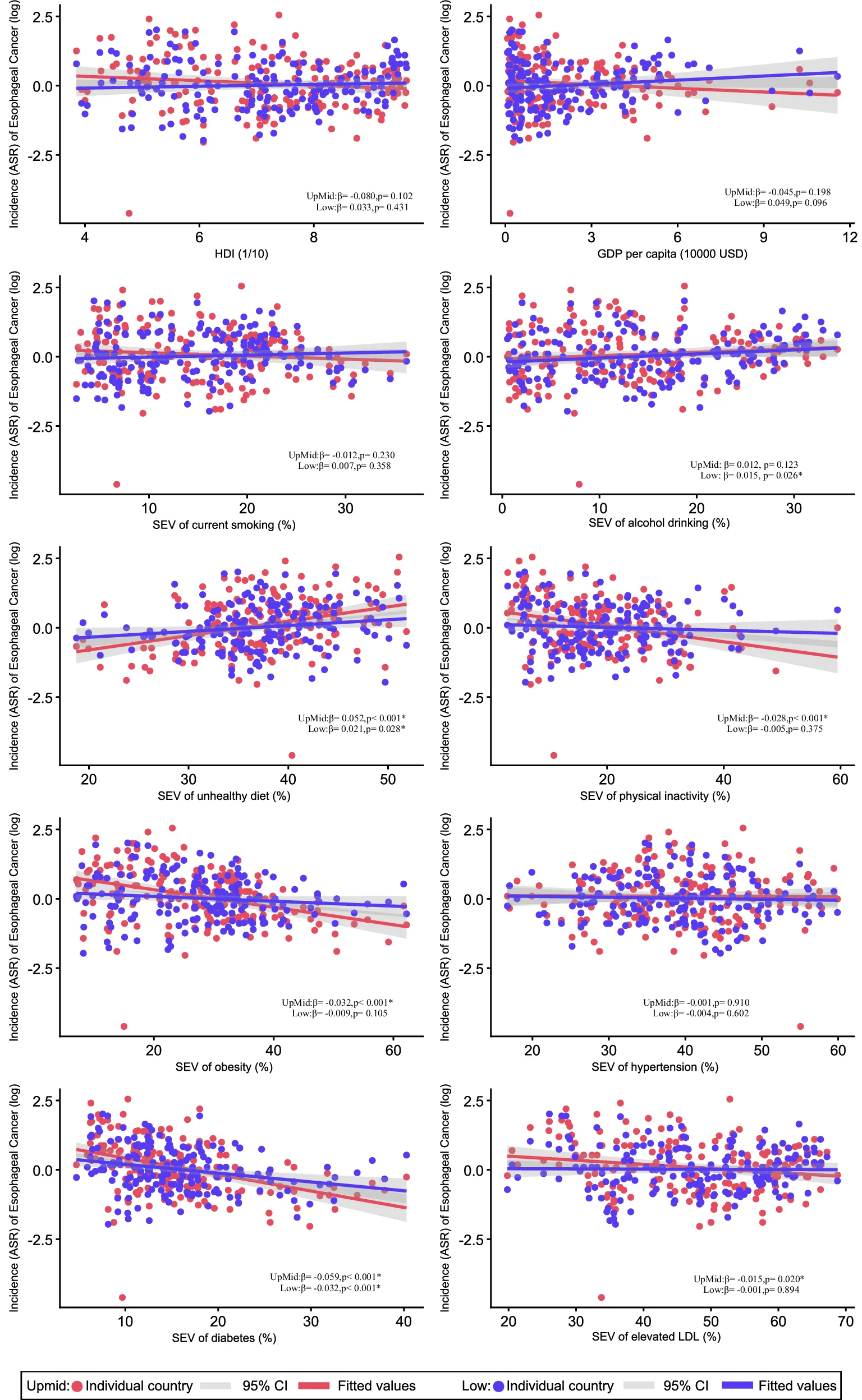
Associations between risk factors and esophageal cancer by anatomical subtype. Associations were evaluated using two-sided linear regression models with log-transformed age-standardized incidence rates as the outcome. Each dot represents an individual country (*n* = 185), and fitted regression lines with 95% confidence intervals are shown. β coefficients and exact P values are annotated in each panel, representing the change in log-transformed age-standardized incidence rate per unit increase in each risk-factor indicator. No adjustment was made for multiple comparisons. ASR age-standardized rate, UpMid upper- and middle-third esophageal cancer, Low lower-third esophageal cancer, HDI Human Development Index, GDP Gross Domestic Product, SEV summary exposure value, LDL low-density lipoprotein.

ESCC incidence was positively associated with the prevalence of unhealthy diet (*β* = 0.043, 95% CI 0.021 to 0.065), but negatively associated with HDI (*β* = −0.095, 95% CI −0.189 to −0.001) and the prevalence of physical inactivity (*β* = −0.027, 95% CI −0.041 to −0.013), obesity (*β* = −0.031, 95% CI −0.042 to −0.019), diabetes (*β* = −0.058, 95% CI −0.078 to −0.039), and lipid disorders (*β* = −0.017, 95% CI −0.029 to −0.004) (Fig. 3, Supplementary Dataset 6). Similar associations were also observed for the UpMid esophageal cancer except for the non-significant association with HDI (Fig. 4). However, the EAC incidence was positively associated with HDI (*β* = 0.211, 95% CI 0.127–0.295), GDP per capita (*β* = 0.163, 95% CI 0.103–0.223), and the prevalence of smoking (*β* = 0.038, 95% CI 0.021–0.054), alcohol drinking (*β* = 0.032, 95% CI 0.018–0.045), physical inactivity (*β* = 0.015, 95% CI 0.001–0.028), obesity (*β* = 0.013, 95% CI 0.002–0.025), and lipid disorders (*β* = 0.024, 95% CI 0.013–0.035). The results of sensitivity analysis using 2012 data were generally consistent, except for GDP per capita was found as a negatively associated factor for ESCC incidence (*β* = −0.083, CI −0.156 to −0.009) (Supplementary Dataset 7). Associations between risk factors and esophageal cancer by sex and age are shown in Supplementary Fig. S1 and Supplementary Fig. S2, respectively.

### Trend of esophageal cancer by region and subtype

ESCC showed a decreasing trend in 13 of 14 regions, while EAC showed an increasing trend in 13 of 14 regions (Supplementary Table 1). Regions that reported the highest increase of EAC were Caribbean (+8.4%), Southern Europe (+5.3%), Western Europe (+4.5%), Sub-Saharan Africa (+2.9%), and South-Eastern Asia (+2.6%). For the anatomical subtype, the UpMid esophageal cancer showed a decreasing trend in 8 of 14 regions, while Low esophageal cancer showed an increasing trend in 8 of 14 regions. The regions that reported the highest increase in Low esophageal cancer were Sub-Saharan Africa (+15.0%), Caribbean (+8.6%), South-Central Asia (+4.4%), Southern Europe (+2.7%), and Western Europe (+1.3%).

### Sex and age-specific trend of esophageal cancer by region and subtype

The rising trend of EAC proportion was more pronounced among females than males and among younger populations aged 15–49 than older populations aged 50–74 (Supplementary Table 1). In females, the highest increase of EAC proportion was reported in Caribbean (+10.5% vs. +7.9% in males), South-Eastern Asia (+5.5% vs. +2.2%), and Sub-Saharan Africa (+3.9% vs. +1.9%). Among younger populations aged 15–49, the highest increase of EAC proportion was reported in Caribbean (+23.7% vs. 8.8% in older populations aged 50–74), Southern Europe (+12.1% vs. +4.9%), and Western Europe (+6.6% vs. +5.6%). For the anatomical subtype, the rising trend of Low esophageal cancer proportion was more pronounced among males than females and among younger populations aged 15–49 than older populations aged 50–74. In males, the highest increase of Low esophageal cancer proportion was observed in Sub-Saharan Africa (+17.8% vs. +10.9% in females). Among younger populations aged 15–49, the highest increase of Low esophageal cancer proportion was in Caribbean (+28.0% vs. +9.0% in older populations aged 50–74).

### Trend of esophageal cancer by country and subtype

**Fig. 5:**
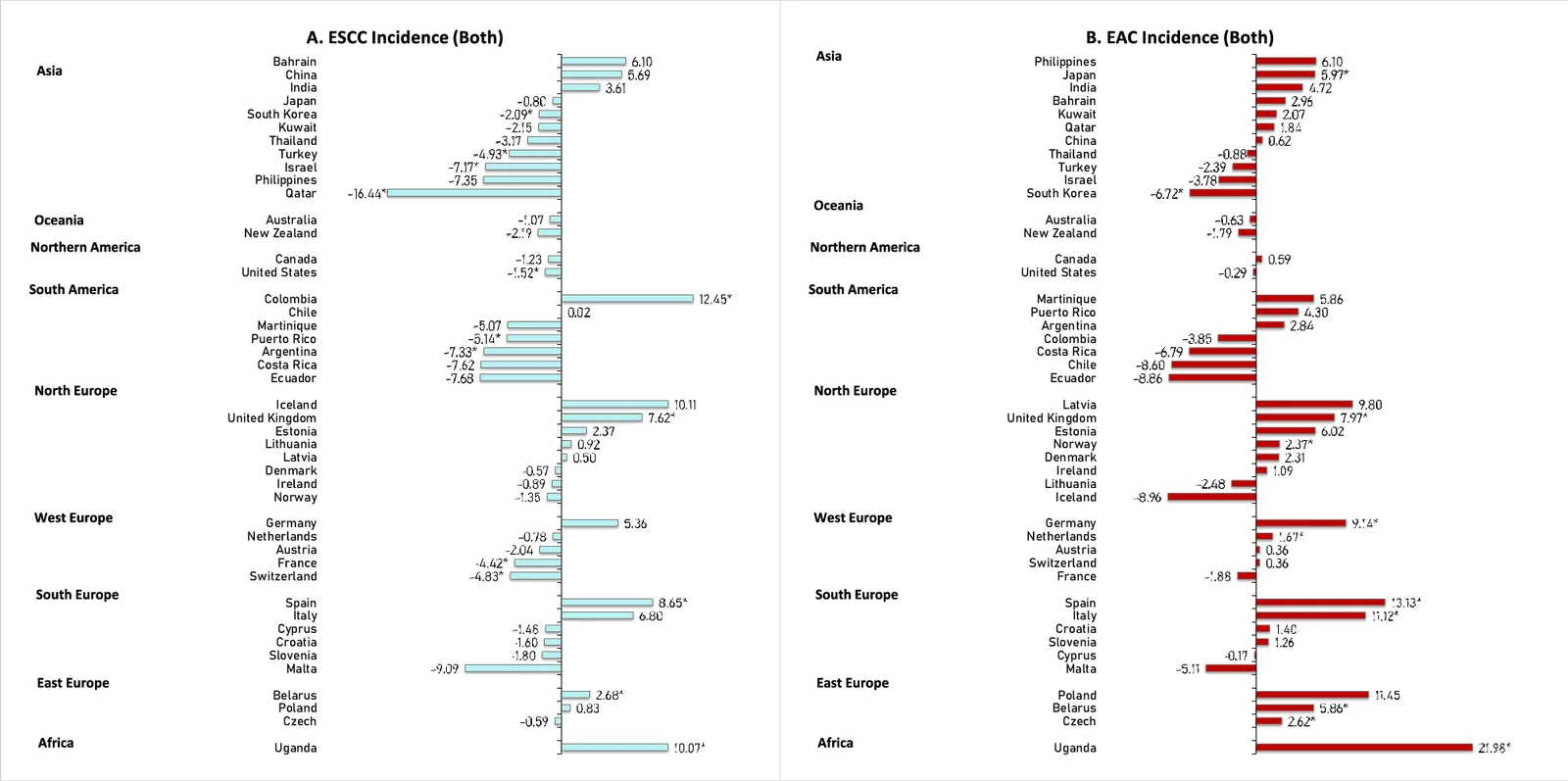
Average annual percentage change of esophageal cancer incidence by histological subtype. **A** shows the AAPC of ESCC incidence from 2008 to 2017 by country (*n* = 45), grouped by region, whereas (**B**) shows the AAPC of EAC incidence from 2008 to 2017 by country (*n* = 45), grouped by region. Asterisks indicate statistically significant trends based on two-sided Joinpoint regression tests (*P* < 0.05). ESCC esophageal squamous cell carcinoma, EAC esophageal adenocarcinoma, AAPC average annual percentage change.

Among the 45 countries included for trend analysis, more countries reported decreasing trends in ESCC (9) than increasing trends (5) (Fig. 5). The most evident increase and decrease were found in Colombia (average annual percentage change [AAPC]: 12.45, 95% CI: 6.00–17.58, *p* < 0.001) and Qatar (AAPC: −16.44, 95% CI: −23.86 to −8.41, *p* < 0.001), respectively (Supplementary Dataset 8). The opposite was found for EAC, in which 10 countries (highest AAPC: Uganda: 21.98, 95% CI: 6.32–39.62, *p* = 0.005) showed an increasing trend, but only South Korea (AAPC: −6.72, 95% CI: −9.37 to −4.01, *p* < 0.001) reported a significant decreasing trend.

### Age- and sex-specific trend of esophageal cancer by country and subtype

For the incidence trend of esophageal cancer, seven countries showed a significant decreasing trend among males (greatest decrease observed in Ecuador: −8.38, 95% CI: −15.04 to −1.33, *p* = 0.018), and six countries among females (greatest decrease observed in Ecuador: −13.64, 95% CI: −21.25 to −5.59, *p* < 0.001) (Supplementary Fig. S3). By age group, four countries demonstrated a significant decreasing trend among young populations, while five exhibited a significant decline among old populations, with Qatar showing the greatest reduction in both groups (Supplementary Fig. S4).

The incidence trends in ESCC among males and females were largely consistent. More countries reported significant decreasing trends (13 in males, greatest decrease in Qatar: −18.91, 95% CI: −27.39 to −11.60, *p* < 0.001; three in females, greatest decrease in Qatar: −10.32, 95% CI: −18.16 to −1.92, *p* = 0.018) than increasing trends (seven in males; greatest increase observed in Iceland: 18.15, 95% CI: 9.08–28.10, *p* < 0.001; 0 in females) (Supplementary Fig. S5). Among younger populations aged 15–49, more countries exhibited significant increasing trends in ESCC (six countries; greatest increase observed in Iceland: 15.17, 95% CI: 6.02–25.83, *p* < 0.001) compared with decreasing trends (three countries; greatest decrease observed in Denmark: –29.14, 95% CI: −39.89 to −16.75, *p* < 0.001) (Supplementary Fig. S6). On the other hand, older populations aged 50–74 had more countries showing decreasing trends (five countries; greatest decrease observed in Qatar: –21.41, 95% CI: −26.76 to −14.33, *p* < 0.001) than increasing trends (two countries; greatest increase observed in Iceland: 13.38, 95% CI: 1.55–25.99, *p* = 0.020) in ESCC.

Likewise, the incidence trends in EAC among males and females were similar to the overall trend, with significant increasing trends observed in more countries (11 in males, greatest increase observed in Uganda: 24.23, 95% CI: 2.14–50.73, *p* = 0.034; seven in females, greatest increase observed in Poland: 27.72, 95% CI: 22.39–33.14, *p* < 0.001) than decreasing trends (four in males, greatest decrease observed in Ecuador: −11.62, 95% CI: −15.37 to −7.86, *p* < 0.001; two in females, greatest decrease observed in Lithuania: −15.15, 95% CI: −25.51 to −3.48, *p* = 0.013) (Supplementary Fig. S7). In both younger and older populations, more countries demonstrated increasing trends in EAC incidence (11 in younger populations; greatest increase in Italy: 33.41, 95% CI: 11.46–57.94, *p* < 0.001; six in older populations; greatest increase in Puerto Rico: 8.04, 95% CI: 1.35–14.01, *p* = 0.032) than decreasing trends (three in younger populations; greatest decrease in Qatar: –21.22, 95% CI: –23.91 to –18.89, *p* < 0.001; four in older populations; greatest decrease in Ecuador: –12.16, 95% CI: –21.08 to –2.14, *p* = 0.015) (Supplementary Fig. S8).

## Discussion

This study is a comprehensive global analysis of the incidence, risk factors, and trends of esophageal cancer by histological and anatomical subtypes. There are several major findings: (1) there was a remarkable geographical disparity in the incidence of ESCC and EAC, with ESCC and EAC being most prevalent in developing (East and South-Central Asia) and developed regions (namely Northern Europe), respectively; (2) there was an overall decreasing trend in ESCC but an increasing trend in EAC, and these trends were generally consistent across age groups and sexes in both region- and country-specific trend analyses; (3) the incidence of UpMid and Low esophageal cancer differed geographically: the UpMid subtype was more prevalent in developing regions (Eastern Asia) while Low subtype was more common in developed regions (Northern Europe); (4) the temporal trends of UpMid and Low esophageal cancer varied, with UpMid subtype decreasing and Low subtype increasing – a rising trend that was more pronounced in males and younger populations in developing regions (Sub-Saharan Africa and Caribbean).

The incidence of esophageal cancer demonstrated pronounced regional heterogeneity by subtype. ESCC was most prevalent in Eastern Asia and South‑Central Asia, regions collectively known as the Asian esophageal cancer belt, while EAC incidence was highest in Northern Europe and Northern America. Such variation likely reflects distinct etiological profiles, as ESCC is more strongly associated with lifestyle‑related and dietary risk factors. For instance, tea consumption at ≥60 °C was associated with an increased risk of ESCC (HR  =  1.41, 95% CI: 1.10–1.81) compared with <60 °C^18^. Daily intake of 700–999 mL of tea was also linked to higher risk (HR  =  1.59, 95% CI: 1.05–2.40)^18^. The joint exposure to high‑temperature tea and higher consumption volumes further amplified risk, with HRs of 1.95 (95% CI: 1.17–3.25) for 700–1299 mL/day and 1.87 (95% CI: 1.13–3.11) for ≥1300 mL/day^18^. In addition, a review summarizing evidence from China reported that consumption of pickled foods was associated with a 1.76‑fold increased risk of ESCC^19^. Lower intake of protective foods, including fresh fruits, seafood, and milk, has also been associated with increased esophageal cancer risk, suggesting a potential role of poor overall nutrition^20^. Environmental exposures may also contribute in certain high-risk areas; for example, use of deep-well water was inversely associated with ESCC and precursor lesions compared with shallow-well water in a population-based endoscopic survey in Anyang, China^21^. Furthermore, socioeconomic factors such as poverty and low educational attainment have been associated with ESCC but not EAC^22^, which may partly explain the higher incidence of ESCC observed in less developed regions.

In contrast, EAC has been modestly but significantly associated with excess fat consumption and obesity, which are more prevalent in high‑income countries^22^. Obesity may further contribute to the development of key EAC‑related risk factors, including gastroesophageal reflux disease (GERD) and Barrett’s esophagus^22^. Meanwhile, GERD and Barrett’s esophagus primarily affect the distal esophagus, which may help explain why EAC is more closely aligned with Low esophageal cancer than UpMid subtype^23,24^. The overlapping risk factors between Low/EAC subtypes and UpMid/ESCC subtypes may partly explain why the geographical and temporal distribution patterns of UpMid and Low subtypes mirror those observed for ESCC and EAC respectively. A Canadian database study similarly demonstrated parallel trends between Low-EAC and UpMid-ESCC subtypes, with Low subtype incidence mirroring EAC patterns while UpMid tracked ESCC trends ^25^.

Smoking and alcohol consumption have long been established as major risk factors for esophageal cancer, particularly ESCC. A meta‑analysis of 52 studies reported risk ratios of 4.18 (95% CI: 3.42–5.12) for current smokers and 2.05 (95% CI: 1.71–2.45) for former smokers in relation to ESCC, while the corresponding risk ratios for EAC were 2.34 (95% CI: 2.04–2.69) and 1.66 (95% CI: 1.48–1.85), respectively^26^. In a China-focused meta-analysis, alcohol consumption was found to significantly increase the risk of ESCC (OR = 2.00, 95% CI: 1.66–2.40)^27^, but not EAC^28^. Moreover, a review reported a strong synergistic effect between smoking and alcohol use, with combined exposure increasing the relative risk of ESCC by more than 100‑fold compared with smoking or alcohol consumption alone^29^. A previous meta-analysis revealed that diabetes mellitus (DM) was significantly associated with an increased risk of EAC (relative risk = 1.43, 95% CI: 1.35–1.51, *p* < 0.001)^30^. Physical inactivity has also been positively associated with EAC incidence in the present country-level analysis. This may partly reflect broader development-related lifestyle patterns, as the prevalence of physical inactivity was remarkably higher in the most developed countries than in less developed countries (27.8% vs 18.7%) ^31^.

Notably, our country‑level analysis indicated that smoking prevalence was significantly associated with EAC incidence but not with ESCC incidence. In contrast, no significant association was observed between diabetes mellitus prevalence and EAC incidence at the country level. These apparent discrepancies may reflect the ecological nature of the study, as associations observed at the population level do not necessarily correspond to individual‑level risks. In addition to examining risk factors specific to ESCC and EAC, this study also investigated factors associated with the ESCC/EAC ratio to account for situations in which both ESCC and EAC incidence are simultaneously high or low. Assessing risk factors in relation to the ESCC/EAC ratio allows identification of determinants that may influence the relative balance between ESCC and EAC incidence rather than their absolute risks alone. For example, unhealthy diet showed a significant positive association with ESCC but no significant association with EAC. Our analysis of the ESCC/EAC ratio further suggested that unhealthy diet was associated with the relative balance between ESCC and EAC, beyond its direct effect on ESCC.

Our study found that ESCC and UpMid esophageal cancers shared similar risk factors, whereas EAC and Low esophageal cancers were characterized by a distinct but internally consistent set of risk factors. Much of this concordance can be explained by the underlying anatomical and histological distribution of esophageal cancer subtypes. Research has shown that 96% of EAC occurs in the lower esophagus^32–34^, and ESCC is also predominantly located in the upper thoracic (10%) and middle thoracic (58%) regions^34^. Nevertheless, ~32% of ESCC cases occur in the lower thoracic esophagus^34^, indicating that while ESCC generally shares risk factors with UpMid esophageal cancer and EAC shares risk factors with Low esophageal cancer, the overlap in risk factors between these groups cannot be explained solely by anatomical and histological distribution. A previous study reported that ESCC arising in the lower esophagus was characterized by a distinct risk factor profile compared with EAC, including higher prevalence of smoking, alcohol consumption, and gastric mucosal atrophy^35^. In the lower esophagus, ESCC did not exhibit a circumferential distribution, suggesting that its development was less likely to be driven by GERD—a key risk factor commonly associated with EAC^35^. Furthermore, cancer location has prognostic relevance for ESCC, with lower thoracic location contributing to more favorable stage groupings in some tumor-grade combinations^34^. Collectively, these findings underscore the importance of integrating both anatomical and histological information when interpreting the epidemiology and clinical behavior of esophageal cancer.

An increasing trend in EAC incidence was observed in both regional and national analyses. Most countries exhibiting significantly positive AAPCs were developed nations, predominantly located in Europe, including the United Kingdom, Norway, Germany, the Netherlands, Spain, and Italy. This rise in EAC incidence may partly reflect the increasing prevalence of associated risk factors driven by globalization and economic development. For example, the incidence of Barrett’s esophagus has been rising in Europe, with evidence from the Northern Ireland Barrett’s Esophagus Register (NIBR) indicating that its incidence increased by ~159% between 1993 and 2005, from 23.9 to 62.0 cases per 100,000 persons^36^. The true prevalence of Barrett’s esophagus remains uncertain and may be under-characterized because of limited high‑quality epidemiological data in many regions^37^. Another factor that might have contributed to the increasing incidence of EAC is obesity. A survey conducted across 12 European countries reported that nearly half of the population (48.1%, 95% CI: 47.2–49.1) were overweight or obese^38^. This trend is also evident among European children aged 2–13 years, among whom the prevalence of overweight and obesity reached 21.3% (95% CI: 19.2–23.6%) between 2011 and 2016^39^. Furthermore, projections suggest that obesity prevalence among European men will reach maximum levels between 2030 and 2052, while the corresponding peak among women is expected to occur earlier, between 2026 and 2054 ^40^.

In contrast, the improvement of socioeconomic status and declines in smoking prevalence in many settings might have contributed to the decreasing trends of ESCC observed in many regions^41,42^. Reductions in alcohol consumption in some populations may have also contributed, although the available supporting evidence cited here is based on US national alcohol survey data^43^. However, increases in ESCC incidence were noted in certain populations, particularly in sub‑Saharan Africa, especially among younger individuals. This increase may partly reflect substantial rises in smoking prevalence, including a 74.6% increase in the number of smokers in sub-Saharan Africa from 1990 to 2019^42^, together with the continuing importance of smoking initiation among young people^44^. From a molecular and epigenetic perspective, a study of ESCC samples from high-incidence populations in Africa, Asia, and South America revealed that ESCC cases were associated with distinct tumor-specific DNA methylation^45^. PAX9, SIM2, and THSD4 were the top three prioritized genes for replication that demonstrated similar methylation differences between the discovery and replication sets^45^. These findings may warrant further investigation into whether molecular or epigenetic profiles contribute to regional differences in ESCC incidence, although direct evidence is currently lacking. Similar trends with ESCC were found for UpMid esophageal cancer, as alcohol interacts with tobacco in a stronger way in the middle portion of the esophagus, where ESCC occurs more commonly^46^. The trends for both ESCC and EAC were largely consistent across different age groups and between the two sexes.

National screening strategies and diagnostic capacity play a critical role in shaping observed incidence trends of esophageal cancer across countries. China has implemented prevention and screening strategies for esophageal cancer for decades, and population-level endoscopic screening has been ongoing in high-risk areas since 2000^47,48^. Since then, regional screening programs have been progressively refined, including risk-stratified and two-step precision screening approaches^47,49^. Expanded screening may have contributed to increased detection of ESCC; however, because screening efforts are largely focused on high‑incidence regions and high‑risk populations, screening findings have shown higher detection rates among males and older individuals^50^. This may explain the significant AAPC observed among males in China despite a non‑significant overall trend. Because esophageal cancer screening programs in China have primarily targeted ESCC and its precursor lesions, their influence on EAC detection is likely limited. Japan also developed advanced esophageal approaches for ESCC detection. Iodine staining was introduced in Japan in the late 1970s and facilitated detection of superficial and intraepithelial esophageal cancer^51^. In a Japanese health-check cohort, screening endoscopy detected 42 ESCC cases, of which 95.2% were diagnosed at an early stage, highlighting the value of endoscopic capacity for early detection in selected screening settings ^52^.

There are several limitations. First, the limited coverage of the CI5 XII database may affect the accuracy of subtype estimation, particularly in lower‑income countries. Second, this analysis did not generate specific estimates for cervical esophageal cancer, a clinical subtype that may have distinct epidemiological and risk‑factor profiles. Third, due to the ecological design, observed associations between risk factors and incidence cannot be directly inferred at the individual level; nevertheless, the country‑level findings offer valuable insights into how key risk factors relate to esophageal cancer incidence across diverse national contexts and may inform targeted policymaking. Fourth, potential non‑linear associations between risk factors and esophageal cancer could not be fully explored. Finally, progressive changes in cancer registry systems in some countries may limit direct temporal comparisons. Despite these limitations, comparisons across countries, regions, sexes, and age groups within the same time frame are likely to be robust.

Overall, this study highlights substantial global heterogeneity in esophageal cancer incidence by histological and anatomical subtypes. The contrasting patterns of ESCC/UpMid and EAC/Low esophageal cancer suggest that prevention and surveillance strategies should be tailored to locally predominant subtypes and risk-factor profiles. Continued subtype-specific monitoring, particularly by sex and age, will be important for guiding early detection strategies, risk-factor control, and future public health planning.

## Methods

This was an ecological, country-level study using publicly available country-level aggregate data on esophageal cancer incidence and socioeconomic, lifestyle, and metabolic risk-factor indicators. The study included countries available in Global Cancer Observatory (GLOBOCAN) 2022, Cancer Incidence in Five Continents (CI5), United Nations (UN), the World Bank, and Global Health Data Exchange (GHDx) databases, as described below. No individual-level identifiable data were analyzed. This study complied with all relevant ethical regulations and was approved by the Survey and Behavioural Research Ethics Committee, The Chinese University of Hong Kong (No. SBRE-20-332).

### Data sources

The incidence of esophageal cancer was retrieved from the GLOBOCAN^53^, covering 185 countries worldwide in 2022^54^. The estimates in GLOBOCAN were generated from incidence-to-mortality ratios, predictions from trend analyses, and approximation from neighboring nations^53^. The site-specific and histology-specific incidence of esophageal cancer was retrieved from the CI5 XII database^54^, which includes data of 460 cancer registries from 65 countries (Supplementary Dataset 9). The anatomical subtypes of esophageal cancer were defined according to the ICD-10: upper/cervical and upper-third esophageal cancer (C15.0 and C15.3), middle/thoracic and middle-third esophageal cancer (C15.1 and C15.4), lower/abdominal and lower-third esophageal cancer (C15.2 and C15.5), and other and unspecified parts (C15.8-9). The histological subtypes of esophageal cancer were defined according to the third edition of the International Classification of Diseases for Oncology (ICD-O-3): squamous cell carcinoma (ESCC) (8050–8078, 8083–8084), adenocarcinoma (EAC) (8140–8141, 8143–8145, 8190–8231, 8260–8265, 8310, 8401, 8480–8490, 8550–8552, 8570–8574, 8576), unspecified carcinoma (8010–8035), sarcoma (8800–8811, 8830, 8840–8921, 8990–8991, 9040–9044, 9120–9133, 9150, 9540–9581), and unspecified malignant neoplasm (8000–8005). The CI5 XI database was also retrieved to evaluate temporal changes in the proportion of esophageal cancer by subtype^55,56^. The HDI and GDP per capita in 2021 were extracted for each country from the United Nations (UN) and the World Bank^56–59^. The summary exposure values (SEVs), which are risk-weighted prevalences defined by Global Burden of Disease Study (GBD), of common lifestyle and metabolic risk factors, including smoking, alcohol drinking, unhealthy diet, physical inactivity, obesity, hypertension, diabetes, and lipid disorders for each country in 2021 and 2012, were collected from the GHDx 2021 database^56^. Smoking was defined as the prevalence of current smokers and former smokers. Alcohol use was defined as daily consumption of pure alcohol. Dietary risk factor was defined as consumption exceeding the optimal intake of harmful components (red meat, processed meat, sugar sweetened beverages, trans fatty acids, sodium) or falling below the recommended intake of protective components (fruit, vegetables, whole grains, nuts and seeds, fiber, seafood omega-3 fatty acids, omega-6 polyunsaturated fatty, calcium, milk, legumes). Physical activity was measured using metabolic equivalent (MET) minutes of total physical activity per week. Obesity was defined as body mass index (BMI) ≥ 30 kg/m^2^. Hypertension was defined as having a systolic blood pressure (SBP) of 140 mmHg or higher, or a diastolic blood pressure (DBP) of 90 mmHg or higher. Diabetes was defined as having fasting plasma glucose greater than or equal to 126 mg/dL (7 mmol/L) or on treatment. Lipid disorder was defined as elevated low‑density lipoprotein (LDL) cholesterol, measured in mmol/L. More detailed definitions of each risk factor were described in Supplementary Table 2 and in a previous report^60^. The current analysis assumed that the GHDx estimates represented country-level populations comparable to those captured in the outcome databases, all reflecting estimates at the population level for each country. To account for potential latent effects among risk factors, sensitivity analyses were conducted across different time points. The CI5*plus* was assessed for evaluating trend of esophageal cancer incidence by sex, age, and histological subtype across different countries from 2008 to 2017 ^61^.

### Statistics & reproducibility

The variation of esophageal cancer incidence was depicted by choropleth maps, which were created to show its incidence in 2022 by subtype in each country. The World Standard Population was used to generate the age-standardized rates (ASRs) of all estimates^62^. We adopted similar methodologies used by the WHO to estimate the subtype of esophageal cancer. Briefly, sex-specific and age-specific proportions of esophageal cancer subtypes were first calculated for countries with adequate data, considering age groups below and above 50 years to capture subtype distribution differences. National proportions were estimated by aggregating data from multiple cancer registries within a country, and regional proportions were calculated for countries lacking sufficient data by aggregating country-level information. Countries were considered to have ‘sufficient data’ if their cancer registries provided complete and reliable incidence information stratified by sex, age, and subtype. For all others, ‘regional proportions’ were estimated by aggregating available country-level data within corresponding geographic regions. These proportions were then multiplied by total cancer cases by age group, sex, and country from GLOBOCAN 2022 to derive cases and age-standardized incidence rates by sex for each country and region using the direct method and World Standard Population. The percentages of change for each region were calculated and compared between CI5 XII (2013–2017) and CI5 XI (2008–2012) to illustrate the evolving patterns in the proportions of esophageal cancer across different subtypes.

The associations between esophageal cancer incidence (log-transformed age-standardized rate) by subtype, sex, and age for each country and factors, such as HDI, GDP per capita, lifestyle, and metabolic risk factors, were evaluated using linear regression analysis. Beta coefficients (*β*) with 95% CIs were reported to indicate the strength and direction of associations. The *β* estimates refer to the amount of change in the log-transformed ASR of incidence for every unit increase in a predictor variable (e.g., SEV of risk factor). For example, when *β* = 0.010, each unit increase in the predictor (e.g., SEV of smoking) is associated with a 0.010 increase in the log-transformed ASR of incidence. In addition, sensitivity analysis was performed on the 2012 data to evaluate the 10-year latent effect of the risk factors. R version 4.5.1 (R Core Team, 2025) was used to conduct statistical analyses and create figures.

Joinpoint regression was conducted to examine the epidemiological patterns in cancer incidence by subtype over the 10-year period (2008–2017)^63^. The AAPC and its 95% CIs were calculated using the empirical quantile method. The incidence data were logarithmically transformed, and analyses were conducted under the assumption of constant variance with an uncorrelated error model. Weights were assigned to each segment based on their length for the selected duration. The best‑performing model was selected using the weighted Bayesian Information Criterion (BIC) method (Supplementary Dataset 10). For country subgroups with zero incidence for more than eight consecutive years, Joinpoint regression could not be performed, and the results were reported as not applicable (NA). In the statistical tests, *P* values < 0.05 were considered significant. The joinpoint regression was conducted using the Joinpoint Regression Program Version 5.0.2 (National Cancer Institute, Maryland).

### Reporting summary

Further information on research design is available in the Nature Portfolio Reporting Summary linked to this article.

## Supplementary information

Supplementary Information

Description of Additional Supplementary Files

Supplementary Dataset 1

Supplementary Dataset 2

Supplementary Dataset 3

Supplementary Dataset 4

Supplementary Dataset 5

Supplementary Dataset 6

Supplementary Dataset 7

Supplementary Dataset 8

Supplementary Dataset 9

Supplementary Dataset 10

Reporting Summary

Transparent Peer Review file

## Source data

Source Data 1

Source Data 2

## Data Availability

All data used in this study are publicly available from existing repositories and databases. Detailed descriptions of the data sources, access links, and processing procedures are provided in the Methods section and the Appendix. Datasets generated and source data underlying the figures are provided with the paper. Source data are provided with this paper.

## Code availability

Analyses were performed using R version 4.5.1 (R Core Team, 2025) and the analysis code is available from GitHub [https://github.com/JaydenLi59554/Global-Esophageal-Cancer-Subtypes].
