# Supplementary material for: Global incidence of esophageal cancer by histological and anatomical subtypes
Source: Nat Commun. 2026 Sep 1;17:10430. doi: 10.1038/s41467-026-76540-1 (PMC13631342; doi:10.1038/s41467-026-76540-1)
Supplement: Supplementary file 1 [file 41467_2026_76540_MOESM13_ESM.pdf]

nature portfolio

Corresponding author(s): Junjie Huang

Last updated by author(s): Jan 22, 2026

Reporting Summary

Nature Portfolio wishes to improve the reproducibility of the work that we publish. This form provides structure for consistency and transparency in reporting. For further information on Nature Portfolio policies, see our Editorial Policies and the Editorial Policy Checklist.

Statistics

For all statistical analyses, confirm that the following items are present in the figure legend, table legend, main text, or Methods section.

n/a	Confirmed	
☐	☒	The exact sample size (*n*) for each experimental group/condition, given as a discrete number and unit of measurement
☐	☒	A statement on whether measurements were taken from distinct samples or whether the same sample was measured repeatedly
☐	☒	The statistical test(s) used AND whether they are one- or two-sided<br>*Only common tests should be described solely by name; describe more complex techniques in the Methods section.*
☐	☒	A description of all covariates tested
☐	☒	A description of any assumptions or corrections, such as tests of normality and adjustment for multiple comparisons
☐	☒	A full description of the statistical parameters including central tendency (e.g. means) or other basic estimates (e.g. regression coefficient) AND variation (e.g. standard deviation) or associated estimates of uncertainty (e.g. confidence intervals)
☐	☒	For null hypothesis testing, the test statistic (e.g. *F*, *t*, *r*) with confidence intervals, effect sizes, degrees of freedom and *P* value noted<br>*Give P values as exact values whenever suitable.*
☒	☐	For Bayesian analysis, information on the choice of priors and Markov chain Monte Carlo settings
☒	☐	For hierarchical and complex designs, identification of the appropriate level for tests and full reporting of outcomes
☒	☐	Estimates of effect sizes (e.g. Cohen's *d*, Pearson's *r*), indicating how they were calculated

Our web collection on statistics for biologists contains articles on many of the points above.

Software and code

Policy information about availability of computer code

Data collection	The analysis of this study used publicly available summary data from international knowledge bases and previously published sources.
Data analysis	Analyses were performed using R version 4.5.1 (R Core Team, 2025) and codes were available from GitHub [https://github.com/JaydenLi59554/Global-Esophageal-Cancer-Subtypes].

For manuscripts utilizing custom algorithms or software that are central to the research but not yet described in published literature, software must be made available to editors and reviewers. We strongly encourage code deposition in a community repository (e.g. GitHub). See the Nature Portfolio guidelines for submitting code & software for further information.

Data

Policy information about availability of data

All manuscripts must include a data availability statement. This statement should provide the following information, where applicable:

- Accession codes, unique identifiers, or web links for publicly available datasets
- A description of any restrictions on data availability
- For clinical datasets or third party data, please ensure that the statement adheres to our policy

All data used in this study are publicly available from existing repositories and databases. Detailed descriptions of the data sources, access links, and processing procedures are provided in Methods section and the Appendix. Datasets generated and source data underlying the figures are provided with the paper.

Research involving human participants, their data, or biological material

Policy information about studies with human participants or human data. See also policy information about sex, gender (identity/presentation), and sexual orientation and race, ethnicity and racism.

Reporting on sex and gender	The study used publicly available,aggregated"age-sex-specific" data. Sex was treated as a biological variable in the analysis. No individual-level data was collected.
Reporting on race, ethnicity, or other socially relevant groupings	The study does not use race or ethnicity for categorization. The primary groupings are geographical locations.
Population characteristics	This study is an analysis using aggregated public data from 185 countries;it did not recruit human participants. Population characteristics are defined at an aggregate level,including data on demographics and incidence.
Recruitment	No participants were recruited for this study.
Ethics oversight	This study was approved by the Survey and Behavioural Research Ethics Committee, The Chinese University of Hong Kong (No. SBRE-20-332).

Note that full information on the approval of the study protocol must also be provided in the manuscript.

Field-specific reporting

Please select the one below that is the best fit for your research. If you are not sure, read the appropriate sections before making your selection.

☐ Life sciences ☒ Behavioural & social sciences ☐ Ecological, evolutionary & environmental sciences

For a reference copy of the document with all sections, see nature.com/documents/nr-reporting-summary-flat.pdf

Behavioural & social sciences study design

All studies must disclose on these points even when the disclosure is negative.

Study description	This is a quantitative and longitudinal study.
Research sample	The study uses aggregated, publicly available data for 185 countries and territories. Datasets were sourced form the WHO, the World Bank, the United Nations and the Global Health Data Exchange. These data were representative for the respective countries.
Sampling strategy	The study did not use sampling.
Data collection	Data were collected by downloading from on-line databases using excel for this study.
Timing	The study collected data from May 2024 to Jan 2025.
Data exclusions	No data were excluded from the analyses.
Non-participation	Not applicable.
Randomization	Not relevant to the study as there are no experimental groups in this study.

Reporting for specific materials, systems and methods

We require information from authors about some types of materials, experimental systems and methods used in many studies. Here, indicate whether each material, system or method listed is relevant to your study. If you are not sure if a list item applies to your research, read the appropriate section before selecting a response.

Materials & experimental systems

n/a	Involved in the study	
☒	☐	Antibodies
☒	☐	Eukaryotic cell lines
☒	☐	Palaeontology and archaeology
☒	☐	Animals and other organisms
☒	☐	Clinical data
☒	☐	Dual use research of concern
☒	☐	Plants

Methods

n/a	Involved in the study	
☒	☐	ChIP-seq
☒	☐	Flow cytometry
☒	☐	MRI-based neuroimaging

Plants

Seed stocks	Not applicable.
Novel plant genotypes	Not applicable.
Authentication	Not applicable.
