# Supplementary material for: Global incidence of esophageal cancer by histological and anatomical subtypes
Source: Nat Commun. 2026 Sep 1;17:10430. doi: 10.1038/s41467-026-76540-1 (PMC13631342; doi:10.1038/s41467-026-76540-1)
Supplement: Supplementary file 2 [file 41467_2026_76540_MOESM14_ESM.pdf]

nature portfolio

Peer Review File

Global Incidence of Esophageal Cancer by Histological and Anatomical Subtypes

Corresponding Author: Professor Junjie Huang

This file contains all reviewer reports in order by version, followed by all author rebuttals in order by version.

Version 0:

Reviewer comments:

Reviewer #1

(Remarks to the Author)
This manuscript provides a global assessment of the incidence, risk factors, and temporal trends of esophageal cancer by histological and anatomical/location subtypes using data from 186 countries. The topic is of considerable public health relevance, and the large-scale analysis is commendable. However, the manuscript requires substantial revision before it can be considered for publication.
1. Please provide the full names of abbreviations when they first appear, such as SCC and AD.
2. The abstract mentions pathological and anatomical structures, but the Results section refers to "location subtype." Please check and ensure consistent terminology throughout the manuscript.
3. Lines 81–84: "GDP per capita (β = –0.232), and some lifestyle factors, while alcohol drinking (β = 0.015) and dietary factors (β = 0.021) contribute to the increased risk of esophageal cancer occurring at the lower region." What "lower region" refers to.
4. In the Background section, the first three sentences describe the prevalence of esophageal cancer but are not supported by any references. Please add appropriate citations.
5. The logic in the Background section is unclear, and the importance of this study is not well highlighted. To my knowledge, many studies have already examined the disease burden and risk factors for different subtypes of esophageal cancer. It is recommended to better compare with previous studies and emphasize the novelty and significance of the present work.
6. The subtype data were obtained from the CI5 XII database, which has limited coverage. The number of cancer registry sites used for estimation in each country should be clearly stated in a supplementary table. Such estimations may substantially affect data quality, especially when stratified by HDI or GDP.
7. Since the study provides detailed analyses of different subtypes, it is unclear whether the overall SCC/AD indicators are still necessary. If they are retained, their relevance should be clarified in the Discussion.
8. Regarding regional differences in incidence trends, please consider whether certain countries have implemented regional screening programs or other prevention initiatives related to esophageal cancer risk factors, which may have influenced incidence or diagnostic capacity. It is not clear whether the current analytical approach accounts for these long-term contextual changes.
9. In the Discussion, the authors note that SCC and UpMid subtypes share similar risk factors, while Low esophageal and AD subtypes also show similarities. As this represents one of the key findings and potential innovations, could the authors further discuss on the possible clinical or public health implications of the location subtype analysis?
10. It is recommended to add maps showing SCC/AD distribution in Figure 1, which would help visualize the predominant histological subtypes geographically.
11. Please consider adding model fit indicators such as slope, R^2, and p value in Figure 3.
12. It is strongly recommended to include a table of contents or other navigation aids in the supplementary materials, as it is currently difficult to locate specific tables.

Reviewer #2

(Remarks to the Author)
The current manuscript presents noteworthy findings, including a comprehensive global analysis of the incidence and trends of esophageal cancer, stratified by pathological subtype and anatomical site. It also investigates key risk factors associated with the disease. This work offers a valuable update on the global epidemiology of esophageal cancer and contributes

meaningfully to the existing literature. Based on my review, I offer the following comments to help improve the manuscript:

Lines 103–104: The statement that esophageal cancer has only three anatomical subtypes is inaccurate. Cervical esophageal cancer (CEC), though uncommon, accounts for approximately 2%–10% of all esophageal carcinomas. These tumors typically present as locally advanced disease and have a poor 5-year overall survival rate of around 30%. Surgical resection is often not feasible due to the proximity of the tumor to critical structures such as the larynx, trachea, and thyroid gland. The omission of CEC should be acknowledged as a limitation of the current study.

Regarding the central aim of the study—assessing the global disease burden—it is important to note that core metrics typically used in Global Burden of Disease (GBD) studies, such as Disability-Adjusted Life Years (DALYs), were not included. Other essential measures like Quality-Adjusted Life Years (QALYs), healthcare utilization, and economic burden were also absent. While this study adds value to the epidemiologic understanding of esophageal cancer, it does not fulfill the criteria of a comprehensive GBD assessment as outlined by institutions such as IHME and WHO. Therefore, it would be more appropriate to avoid using the term “global burden” in the paper.

In the discussion of incidence trends, the authors may consider incorporating findings from the following reference, which provides insight into key molecular features: Talukdar et al. Genome-Wide DNA Methylation Profiling of Esophageal Squamous Cell Carcinoma from Global High-Incidence Regions Identifies Crucial Genes and Potential Cancer Markers. Cancer Res. 2021 May 15;81(10):2612–2624.

Reviewer #3

(Remarks to the Author)
Global Incidence of Esophageal Cancer by Histological and Anatomical Subtypes: a comprehensive analysis

This is a study based on data from GLOBOCAN looking at the incidence of esophageal cancer in 186 countries. The authors also use another database including information on risk factors and look at ecological associations, which seems to be the main thing done in addition to what is already available from GLOBOCAN. The study is interesting but there are some issues that need to be addressed:

Abstract:

Line 70: ‘by the histological and the anatomical sybtypes as well as the sex-specific...’ would be better as ‘by histological and anatomical subtype as well as sex-specific...’.

Line 76: It is unclear what the model was and therefore what ‘beta’ means here.

Line 77: ‘Joinpoint regression was conducted’ should be ‘joinpoint regression was used’.

Line 79: ‘UpMid’ has not been defined.

Line 80: ‘AD’ and ‘Low’ have not been defined.

Line 81: ‘the older populations’ should be ‘older populations’.

Line 82: It would be clearer if the exposure was written before the outcome, e.g. ‘Lower HDI was associated with higher SCC/AD...’. Also it is unclear what ‘SCC/AD’ means. Is it OR or a ratio?

Lines 90-93: I’m not sure that this follows from this analysis.

Background:

Line 104: ‘were’ should be ‘are’.

Lines 114-115: This could be written more clearly.

Line 124: ‘includes the cancer figures’ needs rewording.

Lines 124-126: A reference is needed.

Line 139: ‘products’ should be ‘product’.

Line 143: Is the Global Health Exchange database representative of the same population as the outcome? If not how is this accounted for in the regressions?

Line 145: ‘it is created’ needs rewording.

Lines 148-149: ‘the study initially computed’ needs rewording.

Lines 152-153: How is ‘sufficient data’ defined? What is meant by ‘regional proportions’?

Lines 159-160: ‘beta coefficients’ is not a method. The following sentence (lines 160-161) is redundant.

Was a linear or Poisson regression used?

Line 164: ‘The Stata’ should be ‘Stata’.

Results:

Line 199: ’UpMid’ has not been defined.

Line 200: Highest and lowest ASR of UpMid?

Line 204: Of Low?

Line 206: Age-standardised incidence of what?

Line 224: ‘old’ should be removed.

Line 225: ‘young’ should be removed.

Line 226: ‘of the old population’ should be replaced by ‘among ages ...’.

Line 228, and throughout: ‘the old and young’ should be replaced by the actual age groups.

Lines 232-234: What are these proportions of?

Lines 237-238, and throughout: ‘CI’ is meaningless. 95% CI?

Line 240: SCC/AD has still not been defined and it is unclear what this term means.

Lines 244-245: ‘unhealthy dietary’ should be reworded. What does it mean?

Lines 266-267, and throughout: subtype nomenclature is not conventional.

Discussion:

Line 322: In 2022 only?

Line 343: What is meant here and why is there a range of hazard ratios?

Line 344: Is ‘led to 95%’ correct?

Lines 346-347: ‘Increased risk’ and similar should be changed to ‘Were associated with a higher risk’ or similar, especially in light of the ecological associations.

Line 352: ‘GERD’ has not been defined.

Line 363: ‘had’ should be ‘have’.

Line 368: There is a redundant space after 2.00.

Lines 369-370: What is meant by ‘would be increased over 100-fold'?

Lines 371-373: How does confounding arise here?

Line 377: ‘DM’ has not been defined.

Lines 379-379: This sentence needs to be rewritten.

Line 380: ‘to significantly increase’ here and throughout implies causality which is not supported by the data.

Line 408: ‘Strength’ should be ‘Strengths’.

Line 410: What does ‘with cancer data’ mean?

Lines 416-418: The ecological nature of the associations is more important, they would have been better assessed at the individual level.

Version 1:

Reviewer comments:

Reviewer #1

(Remarks to the Author)
My comments have been addressed, and I have no further comments.

Reviewer #2

(Remarks to the Author)
The authors have adequately addressed the previously noted limitation regarding the inability to analyze cervical esophageal cancer due to insufficient data granularity and have appropriately acknowledged this issue in the Discussion section. They have revised the manuscript to replace all instances of the term “global burden” with “global incidence,” which more accurately reflects the stated aims of the study; this revision has been consistently applied to the title, study objectives, and main text. In addition, the authors have strengthened the discussion of incidence trends by incorporating the findings of Talukdar et al., as previously recommended. Overall, I did not identify any remaining substantive or inconspicuous errors. I therefore recommend the manuscript for publication in Nature Communications.

Reviewer #3

(Remarks to the Author)
The authors have largely addressed previous comments. It would be good to add more details about how the exposure variables were ascertained and defined. Also the associations of various exposures with incidence are described by betas of which the interpretation is not entirely clear. There are also areas where there are minor grammatical errors.

Version 2:

Reviewer comments:

Reviewer #3

(Remarks to the Author)
Previous comments have been adequately addressed.

Open Access This Peer Review File is licensed under a Creative Commons Attribution 4.0 International License, which permits use, sharing, adaptation, distribution and reproduction in any medium or format, as long as you give appropriate credit to the original author(s) and the source, provide a link to the Creative Commons license, and indicate if changes were made.
In cases where reviewers are anonymous, credit should be given to 'Anonymous Referee' and the source.
The images or other third party material in this Peer Review File are included in the article's Creative Commons license, unless indicated otherwise in a credit line to the material. If material is not included in the article's Creative Commons license and your intended use is not permitted by statutory regulation or exceeds the permitted use, you will need to obtain permission directly from the copyright holder.
To view a copy of this license, visit https://creativecommons.org/licenses/by/4.0/

REVIEWER COMMENTS

Reviewer #1 (Remarks to the Author):

This manuscript provides a global assessment of the incidence, risk factors, and temporal trends of esophageal cancer by histological and anatomical/location subtypes using data from 186 countries. The topic is of considerable public health relevance, and the large-scale analysis is commendable. However, the manuscript requires substantial revision before it can be considered for publication.

Author Response: We sincerely appreciate the positive feedback from the reviewer. We have addressed each comment point-by-point below. We are grateful for the reviewer's insights, which have greatly contributed to the improvement of our manuscript.

1. Please provide the full names of abbreviations when they first appear, such as SCC and AD.

Author Response: Thanks for your comments. We added full names of abbreviations in the abstract and it now reads: 'Results: The incidence of esophageal squamous cell carcinoma (ESCC) (ASR = 7.2) and upper and middle third (UpMid) of esophageal cancer (ASR = 6.0) were most prevalent in Eastern Asia, while esophageal adenocarcinoma (EAC) (ASR = 3.2) and lower third (Low) (ASR = 3.6) were commonest in Northern Europe.'(Page 4, Line 14-17)

2. The abstract mentions pathological and anatomical structures, but the Results section refers to "location subtype." Please check and ensure consistent terminology throughout the manuscript.

Author Response: Thanks for your comments. All location subtype had been converted to anatomical subtype for consistency. For example, it now reads:

'By anatomical subtype, the proportions of esophageal cancer occurring in the upper and middle third (UpMid) and lower third (Low) were 63.4% and 36.6% (Figure 2).' (Page 10, Line 17-18);

'By anatomical subtype, males had a higher ASR of UpMid (4.1 vs. 1.7 in females) and Low (2.7 vs. 1.1) esophageal cancer compared with females.' (Page 11; Line 11-13)

'As for the anatomical subtype, the population aged 50-74 had a higher incidence (UpMid: 12.7 vs. 0.71 in the population aged 15-49; Low: 7.2 vs. 0.52) but the

population aged 15-49 had a higher proportion of Low esophageal cancer (36.3% vs. 42.2% in the population aged 15-49, Supplementary Table 2c).' (Page 12, Line 3-6)

'For the anatomical subtype, the UpMid esophageal cancer showed a decreasing trend in 8 of 14 regions, while Low esophageal cancer showed an increasing trend in 8 of 14 regions.' (Page 13, Line 23; Page 14, Line 1)

'For the anatomical subtype, the rising trend of Low esophageal cancer proportion was more pronounced among males than females; among the young than the old (Figure 5).' (Page 14, Line 12-14)

3. Lines 81–84: "GDP per capita (β = −0.232), and some lifestyle factors, while alcohol drinking (β = 0.015) and dietary factors (β = 0.021) contribute to the increased risk of esophageal cancer occurring at the lower region." What "lower region" refers to.

Our Response: Thanks for your comments. We have refined our expression to avoid misunderstanding, and it now reads: 'Human development index (HDI) (β = -0.338), gross domestic product (GDP) per capita (β = -0.232), smoking (β = -0.055), alcohol drinking (β = -0.028), physical inactivity (β = -0.044), obesity (β = -0.047), diabetes (β = -0.057), and lipid disorder (β = -0.043) were negatively associated with the ESCC to EAC incidence ratio (ESCC/EAC) at a country level, whereas dietary factors (β = 0.037) were positively associated.' (Page 4, Line 18-22)

4. In the Background section, the first three sentences describe the prevalence of esophageal cancer but are not supported by any references. Please add appropriate citations.

Our Response: Thanks for your comments: We have now cited the following reference to support our statements on esophageal cancer prevalence: 'Liu CQ, Ma YL, Qin Q, et al. Epidemiology of esophageal cancer in 2020 and projections to 2030 and 2040. Thorac Cancer 2023; 14(1): 3-11.' (Page 6, Line 1-2)

5. The logic in the Background section is unclear, and the importance of this study is not well highlighted. To my knowledge, many studies have already examined the disease burden and risk factors for different subtypes of esophageal cancer. It is recommended to better compare with previous studies and emphasize the novelty and significance of the present work.

Our Response: Thank you for your comments. We have revised the Background section to provide a more detailed comparison with previous studies and to clearly highlight the research gap. The revised text now reads: 'Prior studies have been done to

explore the burden and risk factors for different subtypes of esophageal cancer, but important research gaps remain as they were limited to a certain population or country[2,11]. Studies from the Global Burden of Disease (GBD), despite offering a global comprehensive analysis, lack insights on subtype differences[12-15]. Furthermore, other existing global subtype analyses are not only outdated but also lack analyses of temporal trends or associated risk factors[16-18]. Therefore, this study aims to fill the gap by providing a comprehensive, up-to-date global analysis of esophageal cancer incidence, risk factors, and temporal trends by subtypes and population subgroups.' (Page 6, Line 16-23)

Newly added reference:

11. Malhotra GK, Yanala U, Ravipati A, Follet M, Vijayakumar M, Are C. Global trends in esophageal cancer. Journal of Surgical Oncology 2017; 115(5): 564-79.

12. Sun L, Zhao K, Liu X, Meng X. Global, regional, and national burden of esophageal cancer using the 2019 global burden of disease study. Scientific Reports 2025; 15(1): 3284.

13. Zhang C, Chen L, Xiu Y, Zhang H, Zhang Y, Ying W. Burden of esophageal cancer in global, regional and national regions from 1990 to 2021 and its projection until 2050: results from the GBD study 2021. Frontiers in Oncology 2025; Volume 14 - 2024.

14. Teng Y, Xia C, Cao M, et al. Esophageal cancer global burden profiles, trends, and contributors. Cancer Biol Med 2024; 21(8): 656-66.

15. Jin W, Huang K, Ding Z, et al. Global, regional, and national burden of esophageal cancer: a systematic analysis of the Global Burden of Disease Study 2021. Biomarker Research 2025; 13(1): 3.

16. Arnold M, Laversanne M, Brown LM, Devesa SS, Bray F. Predicting the Future Burden of Esophageal Cancer by Histological Subtype: International Trends in Incidence up to 2030. Official journal of the American College of Gastroenterology | ACG 2017; 112(8).

17. Lin Y, Wang H-l, Fang K, Zheng Y, Wu J. International trends in esophageal cancer incidence rates by histological subtype (1990–2012) and prediction of the rates to 2030. Esophagus 2022; 19(4): 560-8.

18. Arnold M, Soerjomataram I, Ferlay J, Forman D. Global incidence of oesophageal cancer by histological subtype in 2012. Gut 2015; 64(3): 381-7.

6. The subtype data were obtained from the CI5 XII database, which has limited

coverage. The number of cancer registry sites used for estimation in each country should be clearly stated in a supplementary table. Such estimations may substantially affect data quality, especially when stratified by HDI or GDP.

Our Response: Thank you for your comments. We have added a summary table showing the number of registries by country. In total, 460 registries from 65 countries were included, and the corresponding text in the Methods section has been revised accordingly. It now reads: 'The site-specific and histology-specific incidence of esophageal cancer was retrieved from the Cancer Incidence in Five Continents Volume XII (CI5 XII) database,20 which includes data of 460 cancer registries from 65 countries (Supplementary Table 1).' (Page 7, Line 6-9)

We acknowledge that the limited coverage of the CI5 XII database could affect data quality for subtype estimation, particularly in lower-income countries. It reads: '(1) the limited coverage of the CI5 XII database could affect data quality for subtype estimation, particularly in lower-income countries;' (Page 22, Line 23; Page 23, Line 1)

7. Since the study provides detailed analyses of different subtypes, it is unclear whether the overall SCC/AD indicators are still necessary. If they are retained, their relevance should be clarified in the Discussion.

Our Response: Thank you for your comments. We have retained the overall SCC/AD indicators because they provide a concise summary measure of the relative burden of squamous cell carcinoma versus adenocarcinoma across countries, complementing the more detailed subtype analyses. We agree that clarification in Discussion is needed. The text reads: 'In addition to examining risk factors specific to ESCC and EAC, this study also investigated factors associated with the ESCC/EAC ratio to account for situations in which both ESCC and EAC incidence are simultaneously high or low. Assessing risk factors in relation to the ESCC/EAC ratio allows identification of determinants that may influence the relative balance between ESCC and EAC incidence rather than their absolute risks alone. For example, unhealthy diet showed a significant positive association with ESCC but no significant association with EAC. Our analysis of the ESCC/EAC ratio further confirmed that unhealthy diet influenced the relative balance between ESCC and EAC, beyond its direct effect on ESCC.' (Page 19, Line 2-10)

8. Regarding regional differences in incidence trends, please consider whether certain countries have implemented regional screening programs or other prevention initiatives related to esophageal cancer risk factors, which may have influenced incidence or

diagnostic capacity. It is not clear whether the current analytical approach accounts for these long-term contextual changes.

Our Response: Thank you for your comments. We agree that discussion of prevention initiatives is important, and we have added relevant text. A discussion on existing screening program has been added, and it reads: 'China has implemented its first early detection strategy for esophageal cancer in 1960s through esophageal balloon and sponge cytology[59]. Since then, China has continuously refined and developed regional esophageal screening programs[60,61]. The latest screening method was proposed by Li et al. adopting a two-step process[60]. With advances in screening methods and the expansion of screening programs, more ESCC cases can be detected in China. However, screening efforts are primarily concentrated in high-incidence regions or among high-risk populations, therefore more male positive cases were detected62. This may explain why our study observed a significant AAPC value among males in China, while the overall trend was not significant. Moreover, since screening programs mainly target ESCC, the incidence of EAC has remained relatively unchanged[60]. This highlighted the possibility that observed incidence patterns of esophageal cancer were influenced by diagnostic capacity. Another country that implemented esophageal cancer screening in response to high ESCC incidence was Japan. Unlike China, Japan has iodine staining, the gold standard for detecting ESCC since the late 1970s[63,64]. To date, Japan has remained a global leader in the field of endoscopy, with ample screening resources[64]. In contrast to the rising detection of ESCC in China, the AAPC for ESCC in Japan has remained stable, likely owing to the country's long well-established screening capacity. As a result, few ESCC cases go undetected, and notably, 95.2% of esophageal cancers identified in Japan were diagnosed at an early stage[65]' (Page 22, Line 1-18)

Newly added reference:

59. Li F, Liu M, Guo C, et al. Cost-effectiveness of precision screening for esophageal cancer based on individualized risk stratification in China: real-world evidence from the ESECC trial. Frontiers in Oncology 2022; 12: 1002693.

60. Xia C, Basu P, Kramer BS, et al. Cancer screening in China: a steep road from evidence to implementation. The Lancet Public Health 2023; 8(12): e996-e1005.

61. Chen W, Li H, Zheng R, et al. An initial screening strategy based on epidemiologic information in esophageal cancer screening: a prospective evaluation in a community-based cancer screening cohort in rural China. Gastrointestinal endoscopy 2021; 93(1): 110-8. e2.

62. Li H, Teng Y, Yan X, et al. Profiles and findings of population-based esophageal cancer screening with endoscopy in China: systematic review and meta-analysis. JMIR Public Health and Surveillance 2023; 9(1): e45360.

63. Sano M, Okuda S, Tamura H. Esophagoendoscopy using Lugol's staining: diagnosis of esophageal cancer. Takemoto T, Kawai K, Ida K Chromoendoscopy for the gastrointestinal cancer 1978: 13-24.

64. Ishihara R, Katada C. History of endoscopic diagnosis and treatment for esophageal and pharyngeal squamous cell carcinoma. Digestive Endoscopy 2022; 34(S2): 23-6.

65. Nezu Y, Manabe N, Yoda Y, Haruma K. Effectiveness of screening endoscopy for esophageal squamous cell carcinoma in Japanese males. United European Gastroenterol J 2022; 10(8): 868-73.

9. In the Discussion, the authors note that SCC and UpMid subtypes share similar risk factors, while Low esophageal and AD subtypes also show similarities. As this represents one of the key findings and potential innovations, could the authors further discuss on the possible clinical or public health implications of the location subtype analysis?

Our Response: Thank you for your comments. We have added more arguments in Discussion part, and it reads: 'Our study found that ESCC and UpMid esophageal cancer shared similar risk factors, while EAC and Low esophageal cancer also shared a similar set of risk factors. However, the risk factors for ESCC and UpMid differed from those associated with EAC and Low subtypes. Much of this overlap can be explained by the anatomical and histological distribution. Research has shown that 96% of EAC occurs in the lower esophagus[44,45], and ESCC is also predominantly located in the upper thoracic (10%) and middle thoracic (58%) regions[45]. Nevertheless, approximately 32% of ESCC cases occur in the lower thoracic esophagus[45], indicating that while ESCC generally shares risk factors with the UpMid and EAC shares risk factors with the Low, the overlap in risk factors between these groups cannot be explained solely by anatomical and histological distribution. A study has reported that ESCC in the lower esophagus is characterized by different risk factors compared with EAC, including a higher prevalence of smoking, alcohol consumption, and gastric mucosal atrophy[46]. In the lower esophagus, ESCC does not exhibit a circumferential preference, implying that its development is less likely to be driven by gastroesophageal reflux, a risk factor commonly associated with EAC[46]. Furthermore, a study has demonstrated that lower esophageal ESCC is associated with a more favorable prognosis compared to ESCC located in the upper/middle esophagus[47]. These findings underscored the importance of integrating both anatomical and histological information in understanding esophageal cancer.' (Page 19, Line 12-23; Page 20, Line 1-6)

We have also added more text in the Conclusion to highlight the public health implications of our findings. The revised section now reads: 'ESCC and UpMid subtypes shared similar risk factors, whereas EAC and Low subtypes exhibited a distinct group of

risk factors, underscoring the importance of developing targeted preventive strategies tailored to specific histological and anatomical subtypes of esophageal cancer. Developed economies should consider promoting the prevention and management of GERD and Barrett's esophagus to reduce the incidence of EAC/Low esophageal cancer. On the other hand, developing nations may prioritize reducing tobacco and alcohol consumption and improving obesity management, given their strong influence on ESCC and UpMid esophageal cancer, which are more prevalent in these regions.' (Page 23, Line 17-24; Page 24, Line 1)

44. Rice TW, Rusch VW, Ishwaran H, Blackstone EH, Collaboration WEC. Cancer of the esophagus and esophagogastric junction: data‐driven staging for the seventh edition of the American Joint Committee on Cancer/International Union Against Cancer Cancer Staging Manuals. Cancer 2010; 116(16): 3763-73.

45. Rice TW, Rusch VW, Apperson-Hansen C, et al. Worldwide esophageal cancer collaboration. Diseases of the Esophagus 2009; 22(1): 1-8.

46. Okada M, Ishimura N, Mikami H, et al. Circumferential distribution and clinical characteristics of esophageal cancer in lower esophagus: differences related to histological subtype. Esophagus 2019; 16(1): 98-106.

47. Rice TW, Ishwaran H, Blackstone EH. Oesophageal cancer: location, location, location. European Journal of Cardio-Thoracic Surgery 2015; 48(2): 194-5.

10. It is recommended to add maps showing SCC/AD distribution in Figure 1, which would help visualize the predominant histological subtypes geographically.

Our Response: Thank you for your comments. We have now added a map showing the SCC/AD distribution. Please refer to the revised Figure 1 for further information.

11. Please consider adding model fit indicators such as slope, R^2, and p value in Figure 3.

Our Response: Thank you for your comments. We have revised Figure 3 to include beta coefficients and p values, thereby improving the clarity of the model fit.

12. It is strongly recommended to include a table of contents or other navigation aids in the supplementary materials, as it is currently difficult to locate specific tables.
Our Response: Thank you for your comments. We have added a table of contents with links and page numbers to the supplementary materials to facilitate navigation and make it easier to locate specific tables.

Reviewer #2 (Remarks to the Author):

The current manuscript presents noteworthy findings, including a comprehensive global analysis of the incidence and trends of esophageal cancer, stratified by pathological

subtype and anatomical site. It also investigates key risk factors associated with the disease. This work offers a valuable update on the global epidemiology of esophageal cancer and contributes meaningfully to the existing literature. Based on my review, I offer the following comments to help improve the manuscript:

Author Response: We sincerely appreciate the positive feedback from the reviewer. We have addressed each comment point-by-point below. We are grateful for the reviewer's insights, which have greatly contributed to the improvement of our manuscript.

Lines 103–104: The statement that esophageal cancer has only three anatomical subtypes is inaccurate. Cervical esophageal cancer (CEC), though uncommon, accounts for approximately 2%–10% of all esophageal carcinomas. These tumors typically present as locally advanced disease and have a poor 5-year overall survival rate of around 30%. Surgical resection is often not feasible due to the proximity of the tumor to critical structures such as the larynx, trachea, and thyroid gland. The omission of CEC should be acknowledged as a limitation of the current study.

Author Response: Thanks for your comments regarding the esophageal cancer classification used in our study. In our analysis, the anatomical classification of esophageal cancer was based on ICD-10 codes, as specified in the Methods section: ‘The anatomical subtypes of esophageal cancer were defined according to the ICD-10: upper third (C15.0,3), middle third (C15.1,4), lower third (C15.2,5), and other and unspecified parts (C15.8-9).’ (Page 7, Line 9-11) According to the ICD-10 codes, cases of CEC were coded as C15.0, which had been included in the “upper third (C15.0,3)” category in the CI5 database and our analysis. We recognize the clinical significance of CEC and its potential distinct epidemiological and risk factor profiles.

To address this oversight, we have acknowledged it as a limitation in the discussion section: 'The current analysis did not provide specific estimates for cervical esophageal cancer, which is an important clinical subtype that may have unique epidemiological and risk factor profiles.' We appreciate your valuable contribution to enhancing the clarity and rigor of our manuscript. (Page 23, Line 1-2)

Regarding the central aim of the study—assessing the global disease burden—it is important to note that core metrics typically used in Global Burden of Disease (GBD) studies, such as Disability-Adjusted Life Years (DALYs), were not included. Other essential measures like Quality-Adjusted Life Years (QALYs), healthcare utilization, and economic burden were also absent. While this study adds value to the epidemiologic understanding of esophageal cancer, it does not fulfill the criteria of a comprehensive

GBD assessment as outlined by institutions such as IHME and WHO. Therefore, it would be more appropriate to avoid using the term “global burden” in the paper.

Author Response: Thank you for your valuable comments. We have carefully revised the manuscript, and all instances of the term “global burden” have been replaced with “global incidence” to better reflect the aim of our study. For example, in the Abstract section, the phrasing has been updated accordingly: ‘Background: This study aims to examine the global disease incidence, risk factors, and incidence trends of esophageal cancer by histological and anatomical subtypes as well as sex-specific, age-specific and region-specific incidence of disease with data from 186 countries.’ (Page 4, Line 2-4)

In the discussion of incidence trends, the authors may consider incorporating findings from the following reference, which provides insight into key molecular features: Talukdar et al. Genome-Wide DNA Methylation Profiling of Esophageal Squamous Cell Carcinoma from Global High-Incidence Regions Identifies Crucial Genes and Potential Cancer Markers. Cancer Res. 2021 May 15;81(10):2612–2624.

Author Response: Thank you for your comments. We have expanded our discussion on incidence trends by incorporating the findings of Talukdar et al. The added discussion reads: ‘From a genetic aspect, a research on nine high-incidence countries of Africa, Asia, and South America revealed that ESCC cases were associated with distinct tumor-specific DNA methylation[57]. PAX9, SIM2, and THSD4 were the top three prioritized genes for replication that demonstrated similar methylation difference between the discovery and replication sets57. We may hypothesize that these genetic differences could be linked to the increased incidence of ESCC in these regions, although direct evidence is currently lacking. Further studies are therefore warranted to elucidate this association and help unravel the underlying mechanisms. (Page 21, Line 12-19)

Newly added reference:

57. Talukdar FR, Soares Lima SC, Khoueiry R, et al. Genome-Wide DNA Methylation Profiling of Esophageal Squamous Cell Carcinoma from Global High-Incidence Regions Identifies Crucial Genes and Potential Cancer Markers. Cancer Res 2021; 81(10): 2612-24.

Reviewer #3 (Remarks to the Author):

Global Incidence of Esophageal Cancer by Histological and Anatomical Subtypes: a comprehensive analysis

This is a study based on data from GLOBOCAN looking at the incidence of esophageal

cancer in 186 countries. The authors also use another database including information on risk factors and look at ecological associations, which seems to be the main thing done in addition to what is already available from GLOBOCAN. The study is interesting but there are some issues that need to be addressed:

Author Response: We sincerely appreciate the positive feedback from the reviewer. We have addressed each comment point-by-point below. We are grateful for the reviewer's insights, which have greatly contributed to the improvement of our manuscript.

Abstract:

Line 70: ‘by the histological and the anatomical sybtypes as well as the sex-specific...’ would be better as ‘by histological and anatomical subtype as well as sex-specific...’.
Author Response: Thanks for your comments. We have revised accordingly, and it now reads: ‘Background: This study aims to examine the global disease incidence, risk factors, and incidence trends of esophageal cancer by histological and anatomical subtypes as well as sex-specific, age-specific and region-specific incidence of disease with data from 186 countries.’ (Page 4, Line 2-4)

Line 76: It is unclear what the model was and therefore what ‘beta’ means here.
Author Response: Thanks for your comments. We used the linear regression analysis, and added a brief introduction on what ‘beta’ means. It now reads: ‘The weighted prevalence of risk factors for each country was obtained and its associations with incidence by subtypes were measured using beta coefficients (β) with 95% confidence intervals (CI) utilizing linear regression analysis. β coefficients represented the change in the log-transformed ASR of incidence for each unit increase in a predictor variable.’ (Page 4, Line 7-11)

Line 77: ‘Joinpoint regression was conducted’ should be ‘joinpoint regression was used’.
Author Response: Thanks for your comments. We have adjusted accordingly, and it now reads: ‘Joinpoint regression was used for the evaluation of epidemiologic trends.’ (Page 4, Line 11-12)

Line 79: ‘UpMid’ has not been defined, and Line 80: ‘AD’ and ‘Low’ have not been defined.
Author Response: Thanks for your comments. We added full names of abbreviations in the abstract and it now reads: ‘Results: The incidence of esophageal squamous cell carcinoma (ESCC) (ASR = 7.2) and upper and middle third (UpMid) of esophageal cancer (ASR = 6.0) were most prevalent in Eastern Asia, while esophageal

adenocarcinoma (EAC) (ASR = 3.2) and lower third (Low) (ASR = 3.6) were the commonest in Northern Europe.' (Page 4, Line 14-17)

Line 81: 'the older populations' should be 'older populations'.
Author Response: Thanks for your comments, and we have revised accordingly: 'A higher incidence was found among males and older populations.' (Page 4, Line 17-18)

Line 82: It would be clearer if the exposure was written before the outcome, e.g. 'Lower HDI was associated with higher SCC/AD...'. Also it is unclear what 'SCC/AD' means. Is it OR or a ratio?
Author Response: Thank you for your helpful comments. We have clarified the definition of SCC/AD, specifying that it refers to the ratio of SCC to AD incidence. In addition, we revised the sentence so that exposures are presented before the outcome. The text now reads: "Human development index (HDI) (β = -0.338), gross domestic product (GDP) per capita (β = -0.232), smoking (β = -0.055), alcohol drinking (β = -0.028), physical inactivity (β = -0.044), obesity (β = -0.047), diabetes (β = -0.057), and lipid disorder (β = -0.043) were negatively associated with the ESCC to EAC incidence ratio (ESCC/EAC) at a country level, whereas dietary factors (β = 0.037) were positively associated. (Page 4, Lines 18-22).

Lines 90-93: I'm not sure that this follows from this analysis.
Author Response: Thank you for your comments. We have revised our sentences to strengthen the underlying logic and explain why different actions are recommended. The revised sentence now reads: 'The disease incidence, risk factors, and trends of esophageal cancer varied significantly by histological and anatomical subtypes in different regions and countries. EAC and the Low subtype generally shared risk factors and exhibited increasing trends; in contrast, ESCC and the UpMid subtype shared a different risk profile and showed an overall decreasing trend. In developed regions, EAC and Low subtypes were more common, whereas in developing regions, ESCC and UpMid subtypes predominated. Our findings may assist developing tailored prevention strategies based on local emerging esophageal cancer subtypes and relevant risk factors.' (Page 4, Lines 26–30; Page 5, Lines 1-2).

Background:

Line 104: 'were' should be 'are'.
Author Response: Thank you for your comments, and it has been revised according: 'Upper, Middle and Lower esophageal cancer. ESCC and EAC are distinctively different from each other in terms of epidemiology, risk factors and prognosis.' (Page 6, Line 6-7)

Lines 114-115: This could be written more clearly.

Author Response: Thank you for your comments. We have rewritten this sentence to improve its clarity. It now reads: 'For instance, both subtypes are associated with smoking and low fruit and vegetable consumption, while obesity, Barrett's esophagus, and gastroesophageal reflux disease are linked specifically to EAC, and alcohol consumption is linked specifically to ESCC.10.' (Page 6, Line 12-15)

Line 124: 'includes the cancer figures' needs rewording.

Author Response: Thank you for your comments, and it has been revised to be more clear: 'The incidence of esophageal cancer was retrieved from The Global Cancer Observatory (GLOBOCAN),[19] covering 185 countries worldwide in 2022.' (Page 7, 3-4)

Lines 124-126: A reference is needed.

Author Response: Thank you for your comments. We have added the following reference to clarify how the GLOBOCAN estimates were generated: Bray F, Laversanne M, Sung H, et al. Global cancer statistics 2022: GLOBOCAN estimates of incidence and mortality worldwide for 36 cancers in 185 countries. CA: A Cancer Journal for Clinicians 2024; 74(3): 229-63.

Line 139: 'products' should be 'product'.

Author Response: Thank you for your comments, and it has been revised accordingly: 'The human development index (HDI) and the gross domestic product (GDP) per capita were extracted for each country from the United Nations (UN) and the World Bank.' (Page 7, Line 18-20)

Line 143: Is the Global Health Exchange database representative of the same population as the outcome? If not how is this accounted for in the regressions?

Author Response: Thank you for your thoughtful and important comment. We added clarification in the Method section to explain the question. It reads: 'The GHDx database represented the same population as the outcome databases, all reflecting national estimates at the population level for each country. To further account for potential latent effects among risk factors, sensitivity analyses were conducted across different time points, and the results remained consistent.' (Page 8, Line 1-4)

Line 145: 'it is created' needs rewording.

Author Response: Thank you for your comments. It has been revised to: 'The variation of esophageal cancer incidence was depicted by choropleth maps, which were created for showing its incidence in 2022 by subtypes in each country.' (Page 8, Line 7-8)

Lines 148-149: 'the study initially computed' needs rewording.
Author Response: Thank you for your comments, and we have revised this sentence to make it clearer: 'Briefly, sex-specific and age-specific proportions of esophageal cancer subtypes were first calculated for countries with adequate data, considering age groups below and above 50 years to capture subtype distribution differences.' (Page 8, Line 10-13)

Lines 152-153: How is 'sufficient data' defined? What is meant by 'regional proportions'?
Author Response: Thank you for your comments. We added definitions about 'sufficient data' and 'regional proportions' in the main text. It now reads: 'Countries were considered to have 'sufficient data' if their cancer registries provided complete and reliable incidence information stratified by sex, age, and subtype. For all others, 'regional proportions' were estimated by aggregating available country-level data within corresponding geographic regions'. (Page 8, Line 15-18)

Lines 159-160: 'beta coefficients' is not a method. The following sentence (lines 160-161) is redundant.

Author Response: Thank you for your comments. We have made further clarification on method used, and refined our sentences to avoid redundancy. The sentence now reads: 'The relationship of esophageal cancer incidence (log transformation) by subtype, sex, and age for each country with factors, such as HDI, GDP per capita, lifestyle, and metabolic risk factors, were evaluated using linear regression analysis. Beta coefficients (β) with related 95% confidence intervals (CIs) were reported to indicate the strength and direction of associations.' (Page 9, Line 1-4)

Was a linear or Poisson regression used?
Author Response: Thank you for your comments. We have revised the text to clarify the method used. The sentence now reads: 'The relationship of esophageal cancer incidence (log transformation) by subtype, sex, and age for each country with factors, such as HDI, GDP per capita, lifestyle, and metabolic risk factors, were evaluated using linear regression analysis. Beta coefficients (β) with related 95% confidence intervals (CIs) were reported to indicate the strength and direction of associations.' (Page 9, Line 1-4)

Line 164: 'The Stata' should be 'Stata'.
Author Response: Thank you for your comments, and we have revised the issue

accordingly: 'Stata 16.0 (StataCorp, College Station, Texas) was used to conduct statistical analyses and create figures.' (Page 9, Line 7-8)

Results:

Line 199: 'UpMid' has not been defined.
Author Response: Thank you for your comments. 'UpMid' was defined in the previous sentence, and it reads: 'By anatomical subtype, the proportions of esophageal cancer occurring in the upper and middle third (UpMid) and lower third (Low) were 63.4% and 36.6% (Figure 2).' (Page 10, Line 17-18)

Line 200: Highest and lowest ASR of UpMid?
Author Response: Thank you for your comments. The sentence has been rewritten to avoid ambiguity. The sentence now reads: 'The highest and lowest ASR of UpMid were observed in Eastern Asia (6.0) and Northern Africa (0.59), indicating a tenfold difference between regions.' (Page 10, Line 19-21)

Line 204: Of Low?
Author Response: Thank you for your comments. The sentence has been rewritten to avoid ambiguity. The sentence now reads: 'The highest and lowest ASR of Low were observed in Northern Europe (3.6) and Southern Europe (0.79), indicating a four-fold difference between regions.' (Page 11, Line 1-2)

Line 206: Age-standardised incidence of what?
Author Response: Thank you for your comments. The sentence has been revised to be clearer: 'The highest age-standardized incidence rate of esophageal cancer in males was found in Eastern Asia (12.2 vs. 3.4 in females), Northern Europe (7.6 vs. 2.6), and South-Central Asia (7.3 vs. 4.2).' (Page 11, Line 7-9)

Line 224: 'old' should be removed.
Author Response: Thank you for your comments. The sentence has been revised: 'In the population aged 50-74, the ASR of esophageal cancer was 19.8 (334,899), which was higher than in the population aged 15-49 (1.1, 45,242) (Supplementary Table 2c).' (Page 11, Line 20-21)

Line 225: 'young' should be removed.
Author Response: Thank you for your comments. The sentence has been revised: 'In the population aged 50-74, the ASR of esophageal cancer was 19.8 (334,899), which was higher than in the population aged 15-49 (1.1, 45,242) (Supplementary Table 2c).'

(Page 11, Line 20-21)

Line 226: ‘of the old population’ should be replaced by ‘among ages ...’.
Author Response: Thank you for your comments. The sentence has been revised: ‘The highest incidence of the population aged 50-74 was found in Eastern Asia (31.8 vs. 0.67 in the population aged 15-49), South-Central Asia (21.6 vs. 2.3), and Northern Europe (20.2 vs. 0.62).’ (Page 11, Line 21-23)

Line 228, and throughout: ‘the old and young’ should be replaced by the actual age groups.
Author Response: Thank you for your comments. We have replaced the terms “the old and young” with the specific age groups used in our analysis. It now reads: ‘In the population aged 50-74, the ASR of esophageal cancer was 19.8 (334,899), which was higher than in the population aged 15-49 (1.1, 45,242) (Supplementary Table 2c). The highest incidence of the population aged 50-74 was found in Eastern Asia (31.8 vs. 0.67 in the population aged 15-49), South-Central Asia (21.6 vs. 2.3), and Northern Europe (20.2 vs. 0.62). The population aged 15-49 had a slightly higher proportion of ESCC and lower proportion of EAC compared to the population aged 50-74 (ESCC: 87.3% in the population aged 15-49 vs. 83.8% in the population aged 50-74; EAC: 11.9% vs. 14.8% , Supplementary Table 2c). As for the anatomical subtype, the population aged 50-74 had a higher incidence (UpMid: 12.7 vs. 0.71 in the population aged 15-49; Low: 7.2 vs. 0.52) but the population aged 15-49 had a higher proportion of Low esophageal cancer (36.3% vs. 42.2% in the population aged 15-49, Supplementary Table 2c). The highest proportion of Low esophageal cancer in the population aged 15-49 was found in Oceania (79.2% in the population aged 15-49 vs. 68.9%), Northern America (78.8% vs. 73.7%), and Caribbean (75.9% vs. 48.3%).’ (Page 11, Line 20-23; Page 12, Line 1-9)

Lines 232-234: What are these proportions of?
Author Response: Thank you for your comments. These proportions of Low esophageal cancer refer to esophageal cancer occurred in lower third part. We have refine our main text to clarify, and it reads: ‘As for the anatomical subtype, the population aged 50-74 had a higher incidence (UpMid: 12.7 vs. 0.71 in the population aged 15-49; Low: 7.2 vs. 0.52) but the population aged 15-49 had a higher proportion of Low esophageal cancer (36.3% vs. 42.2% in the population aged 15-49, Supplementary Table 2c).’ (Page 12, Line 3-6)

‘Low esophageal cancer’ was defined earlier in the paragraph: ‘By anatomical subtype, the proportions of esophageal cancer occurring in the upper and middle third (UpMid) and lower third (Low) were 63.4% and 36.6% (Figure 2).’ (Page 10, Line 17-18)

Lines 237-238, and throughout: 'CI' is meaningless. 95% CI?
Author Response: Thank you for your comments. We have revised this throughout the manuscript, specifying that all 'CI' actually refers to 95% CI. And it now reads: 'Esophageal cancer incidence was positively associated with alcohol drinking (β=0.016, 95% CI 0.001 to 0.030) and dietary risk factors (β=0.035, 95% CI 0.015 to 0.056), but negatively associated with prevalences of physical inactivity (β=-0.014, 95% CI -0.027 to -0.001), obesity (β=-0.018, 95% CI -0.030 to -0.007), and diabetes (β=-0.043, 95% CI -0.062 to -0.024) at a country level.' (Page 12, Line 12-16)

Line 240: SCC/AD has still not been defined and it is unclear what this term means.
Author Response: Thank you for your comments. We have clarified the definition of SCC/AD in the text, and it now reads: 'ESCC to EAC esophageal cancer incidence ratio (ESCC/EAC) was positively associated with unhealthy diet (β=0.037, 95% CI 0.016 to 0.059; Supplementary Table 3a), but negatively associated with HDI (β=-0.338, 95% CI -0.417 to -0.258), GDP per capita (β=-0.232, 95% CI -0.290 to -0.174), and prevalences of smoking (β=-0.055, 95% CI -0.072 to -0.038), alcohol drinking (β=-0.028, 95% CI -0.042 to -0.013), physical inactivity (β=-0.044, 95% CI -0.057 to -0.031), obesity (β=-0.047, 95% CI -0.057 to -0.036), diabetes (β=-0.057, 95% CI -0.076 to -0.037), and lipid disorder (β=-0.043, 95% CI -0.054 to -0.033) at a country level)' (Page12, Line 16-22)

Lines 244-245: 'unhealthy dietary' should be reworded. What does it mean?
Author Response: Thank you for your comments. We added definition in the Method section, and it reads: 'Unhealthy diet was defined as the consumption of more than the optimal level for harmful items (e.g sodium) or less than the optimal level for protective items (e.g. fruits) according to the GHDx database.' (Page 7, Line23-24; Page 8, Line 1)

Lines 266-267, and throughout: subtype nomenclature is not conventional.
Author Response: Thank you for your comments. In response, we have revised the manuscript to use standard nomenclature for histological subtypes. Specifically, " squamous cell carcinoma (SCC)" and " esophageal adenocarcinoma (AD)" have been changed to "esophageal squamous cell carcinoma (ESCC)" and "esophageal adenocarcinoma (EAC)" throughout the text. For example, it reads: 'The histological subtypes of esophageal cancer were defined according to the third edition of the International Classification of Diseases for Oncology (ICD-O-3): squamous cell carcinoma (ESCC) (8050–8078, 8083–8084), adenocarcinoma (EAC) (8140–8141,

8143–8145, 8190–8231, 8260–8265, 8310, 8401, 8480–8490, 8550–8552, 8570–8574, 8576),' (Page 7, Line 11-14)

Discussion:
Line 322: In 2022 only?
Author Response: Thank you for your comments. It is not limited to 2022 but observed across multiple years. We have revised the text accordingly, and it now reads: 'Disease incidence of esophageal cancer and its subtype varied significantly among different regions.' (Page 16, Line 18-19)

Line 343: What is meant here and why is there a range of hazard ratios?
Line 344: Is 'led to 95%' correct?

Author Response: Thank you for your comments. The original expression was unclear, and we have revised it for clarity. It now reads: 'For instance, drinking tea at ⩾60 °C was associated with a hazard ratio (HR) of 1.41 (95% CI: 1.10–1.81) compared to <60 °C29. Consuming 700–999 mL of tea (black or green) per day was linked to an HR of 1.59 (95% CI: 1.05–2.40) compared to drinking less than 700 mL29. The combination of drinking hot tea at ⩾60 °C and consuming 700–1,299 mL/day was associated with a higher HR of 1.95 (95% CI: 1.17–3.25) for ESCC, whereas combination of drinking hot tea at ⩾60 °C and drinking ⩾1,300 mL/day was linked to an HR of 1.87 (95% CI: 1.13–3.11).' (Page 16, Line 23-24; Page 17, Line 1-5)
Lines 346-347: 'Increased risk' and similar should be changed to 'Were associated with a higher risk' or similar, especially in light of the ecological associations.
Author Response: Thank you for your comments. It is true that these results suggested associations, and we should be more careful with our wording. The text has been revised and now reads: 'The consumption of pickled food was associated with a 1.76-fold higher risk of ESCC.30 Poor nutrition such as lack of protein and carbohydrates was significantly associated with an increased risk of EC.31' (Page 17, Line 5-7)

Line 352: 'GERD' has not been defined.
Author Response: Thank you for your comments. We have revised the text to define GERD. It now reads: 'while obesity potentially leads to the salient risk factors of EAC: gastroesophageal reflux disease (GERD) and Barrett's esophagus.' (Page 17, Line 13-14)

Line 363: 'had' should be 'have'.

Author Response: Thank you for your comments. We have revised the text. It now reads: 'Smoking and alcohol drinking have long been established as major risk factors of esophageal cancer, in particular ESCC.' (Page 18, Line 2-3)

Line 368: There is a redundant space after 2.00.

Author Response: Thank you for your comments. We have revised and it now reads: 'Alcohol consumption significantly increased the risk of ESCC (OR: 2.00, 95% CI: 1.66 to 2.40),' (Page 18, Line 6-7)

Lines 369-370: What is meant by 'would be increased over 100-fold'?

Author Response: Thank you for your comments. We have refined the sentence to improve readability. It now reads: 'Also, a synergistic effect between smoking and drinking has been reported – combined exposure can increase the relative risk of ESCC by more than 100-fold compared with smoking or drinking alone.' (Page 18, Line 8-10)

Lines 371-373: How does confounding arise here?

Author Response: Thank you for your comments. The nonsignificant association between smoking and ESCC might be due to the ecological nature of this study. We have added corresponding explanation in the main text, and it reads: 'However, our findings suggested that prevalence of smoking was only significantly associated with EAC, but not significantly related to ESCC incidence at a country level. This disparity might be due to the ecological nature of this study, which may not reflect true relationship at the individual level.' (Page 18, Line 10-13)

Line 377: 'DM' has not been defined.

Lines 379-379: This sentence needs to be rewritten.

Author Response: Thank you for your comments. The revised text now reads: 'A previous meta-analysis revealed that diabetes mellitus (DM) was significantly associated with an increased risk of EAC (Relative risk = 1.43, 95% CI 1.35 to 1.51, p<0.001).42 However, at a country-level, our analysis did not find a significant association between diabetes mellitus and EAC incidence.' (Page 18, Line 15-18)

Line 380: 'to significantly increase' here and throughout implies causality which is not supported by the data.

Author Response: Thank you for your comments. We agree that our previous wording improperly implied causality. We have revised the text to emphasize associations only. It now reads: 'Physical inactivity has also been found to be significantly associated with an increased risk of EAC.' (Page 18, Line 18-19)

Line 408: ‘Strength’ should be ‘Strengths’.
Author Response: Thank you for your comments. We have revised the text accordingly: ‘Strengths and limitations’ (Page 22, Line 20)

Line 410: What does ‘with cancer data’ mean?
Author Response: Thank you for your comments. We have revised the text for clarity, and it now reads: ‘This study comprehensively analyzed the disease incidence, risk factors, and the temporal trends of esophageal cancer by subsite and subgroup for 185 countries.’ (Page 22, Line 21-22)

Lines 416-418: The ecological nature of the associations is more important, they would have been better assessed at the individual level.

Author Response: Thank you for your comments. We have revised the text for clarity, and it now reads: ‘given the ecological design of our study, the risk-factor associations may not be applied to the individual level; nevertheless, our country-level analysis revealed how key risk factors were associated with the cancer incidence across distinct national settings and thus inform targeted policymaking’ (Page 23, Line 4-7)

REVIEWER COMMENTS

Reviewer #1 (Remarks to the Author):

My comments have been addressed, and I have no further comments.

Author Response: We sincerely appreciate the positive feedback from the reviewer. We are grateful for the reviewer's previous insights, which have greatly contributed to the improvement of our manuscript.

Reviewer #2 (Remarks to the Author):

The authors have adequately addressed the previously noted limitation regarding the inability to analyze cervical esophageal cancer due to insufficient data granularity and have appropriately acknowledged this issue in the Discussion section. They have revised the manuscript to replace all instances of the term “global burden” with “global incidence,” which more accurately reflects the stated aims of the study; this revision has been consistently applied to the title, study objectives, and main text. In addition, the authors have strengthened the discussion of incidence trends by incorporating the findings of Talukdar et al., as previously recommended. Overall, I did not identify any remaining substantive or inconspicuous errors. I therefore recommend the manuscript for publication in Nature Communications.

Author Response: We sincerely appreciate the positive feedback from the reviewer. We are grateful for the reviewer's previous insights, which have greatly contributed to the improvement of our manuscript.

Reviewer #3 (Remarks to the Author):

The authors have largely addressed previous comments. It would be good to add more details about how the exposure variables were ascertained and defined.

Author Response: Thank you for this helpful comment. We have now added more detailed information on the ascertainment and definitions of the exposure variables in the Methods section. Specifically, we clarified the data sources and operational definitions of each exposure as follows: “*The summary exposure values (risk-weighted prevalence) of common lifestyle and metabolic risk factors, including smoking, alcohol drinking, unhealthy diet, physical inactivity, obesity, hypertension, diabetes, and lipid disorders for each country, were collected from the Global Health Data Exchange (GHDx) database.*[22,26,27] *Smoking was defined as the prevalence of current smokers and former smokers. Alcohol use was defined as daily consumption of pure alcohol. Dietary risk factor was defined as consumption exceeding the optimal intake of harmful*

components (red meat, processed meat, sugar sweetened beverages, trans fatty acids, sodium) or falling below the recommended intake of protective components (fruit, vegetables, whole grains, nuts and seeds, fiber, seafood omega-3 fatty acids, omega-6 polyunsaturated fatty, calcium, milk, legumes). Physical activity was measured using metabolic equivalent (MET) minutes of total physical activity per week. Obesity was defined as body mass index (BMI) ⩾30 kg/m2. Hypertension was defined as having a systolic blood pressure (SBP) of 140 mmHg or higher, or a diastolic blood pressure (DBP) of 90 mmHg or higher. Diabetes was defined as having fasting plasma glucose greater than or equal to 126 mg/dL (7 mmol/L) or on treatment. Lipid disorder was defined as elevated low-density lipoprotein (LDL) cholesterol, measured in mmol/L. More detailed definitions of each risk factor were described in Supplementary Table 2 and in a previous report.[27]" (Page 6: 20-24; Page 7: Line 1-12)

Also the associations of various exposures with incidence are described by betas of which the interpretation is not entirely clear.

Author Response: We appreciate the reviewer's comment and have clarified the interpretation of the beta coefficients in the Methods section. Specifically, we now explain that: "*Beta coefficients (β) with related 95% confidence intervals (CIs) were reported to indicate the strength and direction of associations. The β estimates refer to the amount of change in the outcome variable (ASR of incidence) for every unit increase in a predictor variable (prevalence of risk factor). For example, when β = 0.010, each unit increase in the predictor (e.g., smoking prevalence) is associated with a 0.010 increase in the ASR of incidence.*" (Page 8: Line 14-18)

There are also areas where there are minor grammatical errors.

Author Response: Thank you for noting this. We have carefully reviewed the entire manuscript and revised it to correct grammatical errors and improve overall clarity and readability.
