# Supplementary material for: Global incidence of esophageal cancer by histological and anatomical subtypes
Source: Nat Commun. 2026 Sep 1;17:10430. doi: 10.1038/s41467-026-76540-1 (PMC13631342; doi:10.1038/s41467-026-76540-1)

Table of Contents for Supplementary Materials

Supplementary Table 1. Proportion changes of esophageal cancer incidence by age, sex, and subtype 2

Supplementary Table 2. Risk factor exposure definitions and thresholds as defined by GBD 3

Supplementary Figure 1. Associations between risk factors and esophageal cancer by sex 4

Supplementary Figure 2. Associations between risk factors and esophageal cancer by age 5

Supplementary Figure 3. Annual Average Percentage Change of esophageal cancer incidence by sex 6

Supplementary Figure 4. Annual Average Percentage Change of esophageal cancer incidence by age 7

Supplementary Figure 5. Annual Average Percentage Change of ESCC incidence by sex 8

Supplementary Figure 6. Annual Average Percentage Change of ESCC incidence by age 9

Supplementary Figure 7. Annual Average Percentage Change of EAC incidence by sex 10

Supplementary Figure 8. Annual Average Percentage Change of EAC incidence by age 11

Supplementary Table 1. Proportion changes of esophageal cancer incidence by age, sex, and subtype. Abbreviations: ESCC: esophageal squamous cell carcinoma; EAC: esophageal adenocarcinoma; UpMid: upper and middle-third esophageal cancer; Low: lower-third esophageal cancer

Number	Region	Both Sexes				Male				Female				Young				Old			
		ESCC	EAC	UpMid	Low	ESCC	EAC	UpMid	Low	ESCC	EAC	UpMid	Low	ESCC	EAC	UpMid	Low	ESCC	EAC	UpMid	Low
1	Sub-Saharan Africa	-3.2%	2.9%	-15.0%	15.0%	-1.9%	1.9%	-17.8%	17.8%	-4.6%	3.9%	-10.9%	10.9%	-1.2%	0.9%	1.5%	-1.5%	-3.7%	3.1%	-22.0%	22.0%
2	Northern Africa	0.2%	-0.2%	35.7%	-35.7%	0.9%	-0.9%	22.2%	-22.2%	0.6%	-0.6%	43.3%	-43.3%	18.1%	-18.1%	58.3%	-58.3%	-2.3%	2.3%	36.4%	-36.4%
3	Caribbean	-9.1%	8.4%	-8.6%	8.6%	-8.8%	7.9%	-8.3%	8.3%	-9.8%	10.5%	-9.8%	9.8%	-19.2%	23.7%	-28.0%	28.0%	-9.8%	8.8%	-9.0%	9.0%
4	Latin America	-1.7%	1.4%	4.5%	-4.5%	-1.5%	1.3%	4.4%	-4.4%	-2.1%	1.1%	4.8%	-4.8%	3.0%	-1.5%	11.4%	-11.4%	-0.6%	0.0%	5.5%	-5.5%
5	Northern America	-2.3%	2.0%	-1.0%	1.0%	-2.2%	1.9%	-0.8%	0.8%	-2.7%	2.7%	-1.6%	1.6%	-3.5%	3.5%	-0.9%	0.9%	-2.2%	1.9%	-0.9%	0.9%
6	Eastern Asia	-2.4%	2.2%	4.2%	-4.2%	-2.3%	2.2%	4.9%	-4.9%	-2.7%	2.3%	1.9%	-1.9%	-3.6%	3.3%	1.1%	-1.1%	-2.5%	2.2%	3.8%	-3.8%
7	South-Eastern Asia	-2.6%	2.6%	4.7%	-4.7%	-2.2%	2.2%	5.0%	-5.0%	-5.5%	5.5%	2.1%	-2.1%	-3.5%	3.5%	15.7%	-15.7%	-0.7%	0.8%	2.6%	-2.6%
8	South Central Asia	-0.6%	0.5%	-4.4%	4.4%	-0.5%	0.5%	-4.6%	4.6%	-0.6%	0.5%	-4.0%	4.0%	0.9%	-0.9%	-3.9%	3.9%	-0.8%	0.7%	-4.9%	4.9%
9	Western Asia	-1.0%	1.2%	-1.3%	1.3%	0.4%	-0.2%	0.9%	-0.9%	-2.8%	2.9%	-4.8%	4.8%	0.4%	-0.1%	-1.9%	1.9%	-1.9%	1.4%	-2.8%	2.8%
10	Central and Eastern Europe	-2.0%	2.0%	1.3%	-1.3%	-2.0%	2.0%	0.5%	-0.5%	-1.7%	1.2%	6.2%	-6.2%	-3.2%	2.5%	3.5%	-3.5%	-1.8%	1.8%	0.5%	-0.5%
11	Northern Europe	-1.2%	0.9%	-0.5%	0.5%	-1.0%	0.7%	-0.7%	0.7%	-0.7%	0.4%	0.6%	-0.6%	-0.9%	-0.1%	-0.2%	0.2%	-1.4%	1.4%	-0.5%	0.5%
12	Southern Europe	-5.5%	5.3%	-2.7%	2.7%	-6.8%	6.9%	-4.0%	4.0%	-1.5%	0.2%	1.8%	-1.8%	-12.0%	12.1%	-6.8%	6.8%	-5.2%	4.9%	-4.1%	4.1%
13	Western Europe	-4.7%	4.5%	-1.3%	1.3%	-5.7%	5.4%	-2.0%	2.0%	-1.8%	1.8%	0.8%	-0.8%	-6.9%	6.6%	-5.0%	5.0%	-5.8%	5.6%	-1.6%	1.6%
14	Oceania	-1.3%	0.6%	2.0%	-2.0%	-1.0%	0.0%	1.7%	-1.7%	-1.3%	1.1%	2.6%	-2.6%	3.2%	-3.0%	-1.3%	1.3%	-0.8%	0.2%	2.8%	-2.8%

Supplementary Table 2. Risk factor exposure definitions and thresholds as defined by GBD

**Risk factor**	**Definition of exposure**	**Threshold**
Smoking	Prevalence of current smokers and former smokers	Current smokers: using any smoked tobacco product, either daily or occasionally, at the time of the survey. Former smokers: had ceased using all smoked tobacco products for at least six months, or according to the specific definition adopted in the respective survey
Alcohol use	Daily consumption of pure alcohol	10 g ethanol
Dietary risk factor	Diet low in fruit	340–350 g/day
	Diet low in vegetables	306–372 g/day
	Diet low in whole grains	160–210 g/day
	Diet low in nuts and seeds	19–24 g/day
	Diet low in fibre	22–25 g/day
	Diet low in seafood omega-3 fatty acids	470–660 mg/day
	Diet low in omega6 polyunsaturated fatty acids	9–10% of total daily energy
	Diet low in calcium	0.72–0.86 g/day (males) 1.1–1.2 g/day (females)
	Diet low in milk	280–340 g/day (males) 500–610 g/day (females)
	Diet low in legumes	100–110 g/day
	Diet high in red meat	0–200 g/day
	Diet high in processed meat	0 g/day
	Diet high in sugar sweetened beverages (SSBs)	0 g/day
	Diet high in trans fatty acids	0% of total daily energy
	Diet high in sodium	1–5 g/day
Physical inactivity	Total physical activity per week	< 3600–4400 metabolic equivalent (MET) minutes
Obesity	Body mass index	≥ 30 kg/m^2
Hypertension	Systolic blood pressure or diastolic blood pressure	systolic blood pressure >= 140 mmHg, or diastolic blood pressure >= 90 mmHg or higher
Diabetes	Fasting plasma glucose	fasting plasma glucose >= 126 mg/dL (7 mmol/L) or on treatment
Lipid disorder	Low-density lipoprotein cholesterol	>0.9–1.4 mmol/L

Supplementary Figure 1. Associations between risk factors and esophageal cancer by sex. Associations were evaluated using two-sided linear regression model with log-transformed ASR incidence rate as the outcome (n=185).

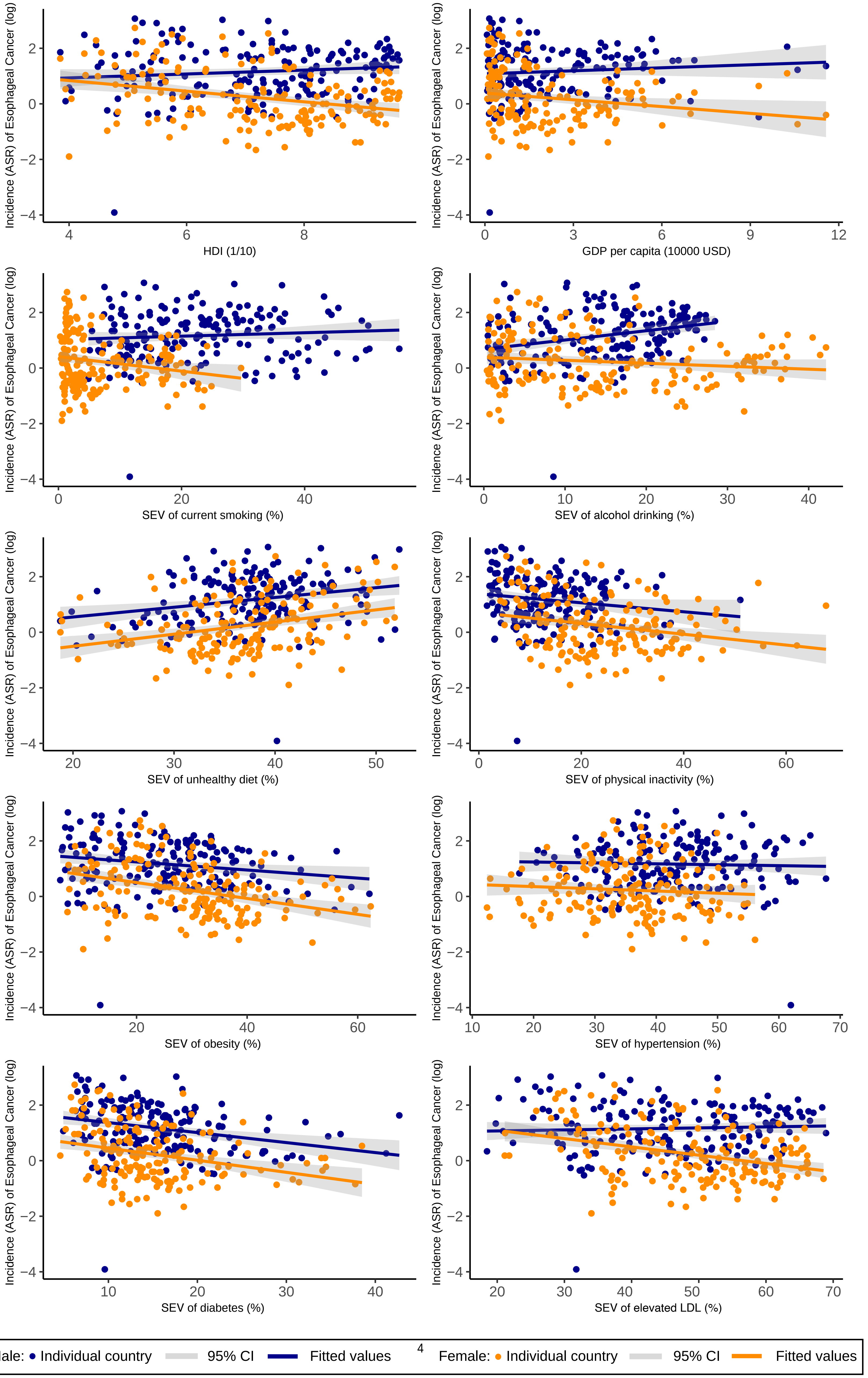

Supplementary Figure 2. Associations between risk factors and esophageal cancer by age. Associations were evaluated using two-sided linear regression model with log-transformed ASR incidence rate as the outcome (n=185).

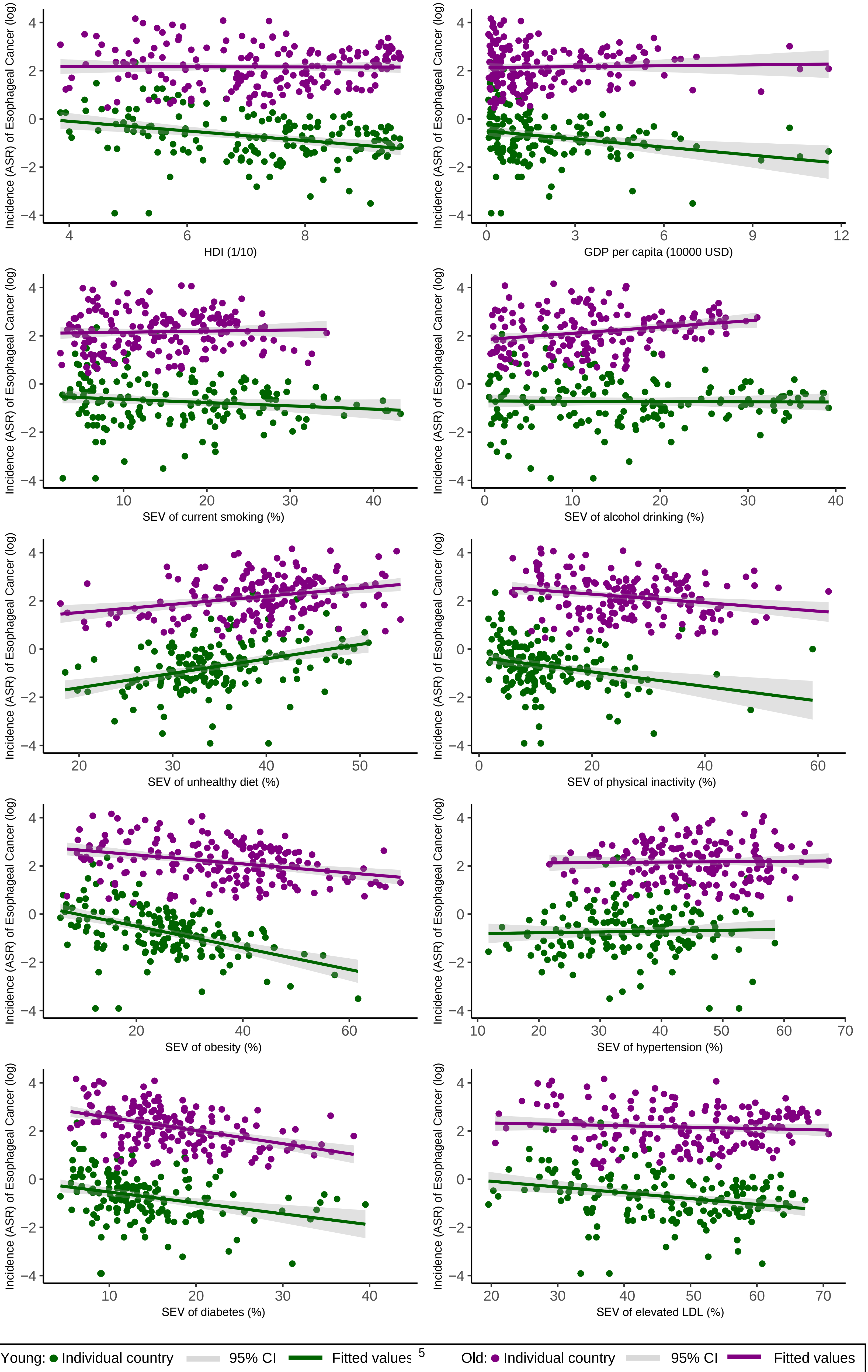

Supplementary Figure 3. Annual Average Percentage Change of esophageal cancer incidence by sex.

From 2008 to 2017 by country (n=45). Two-sided Joinpoint regression test was used. Exact p values were provided in Supplementary Dataset 8, and statistically significant changes ($p < 0.05$) were marked with an asterisk (*).

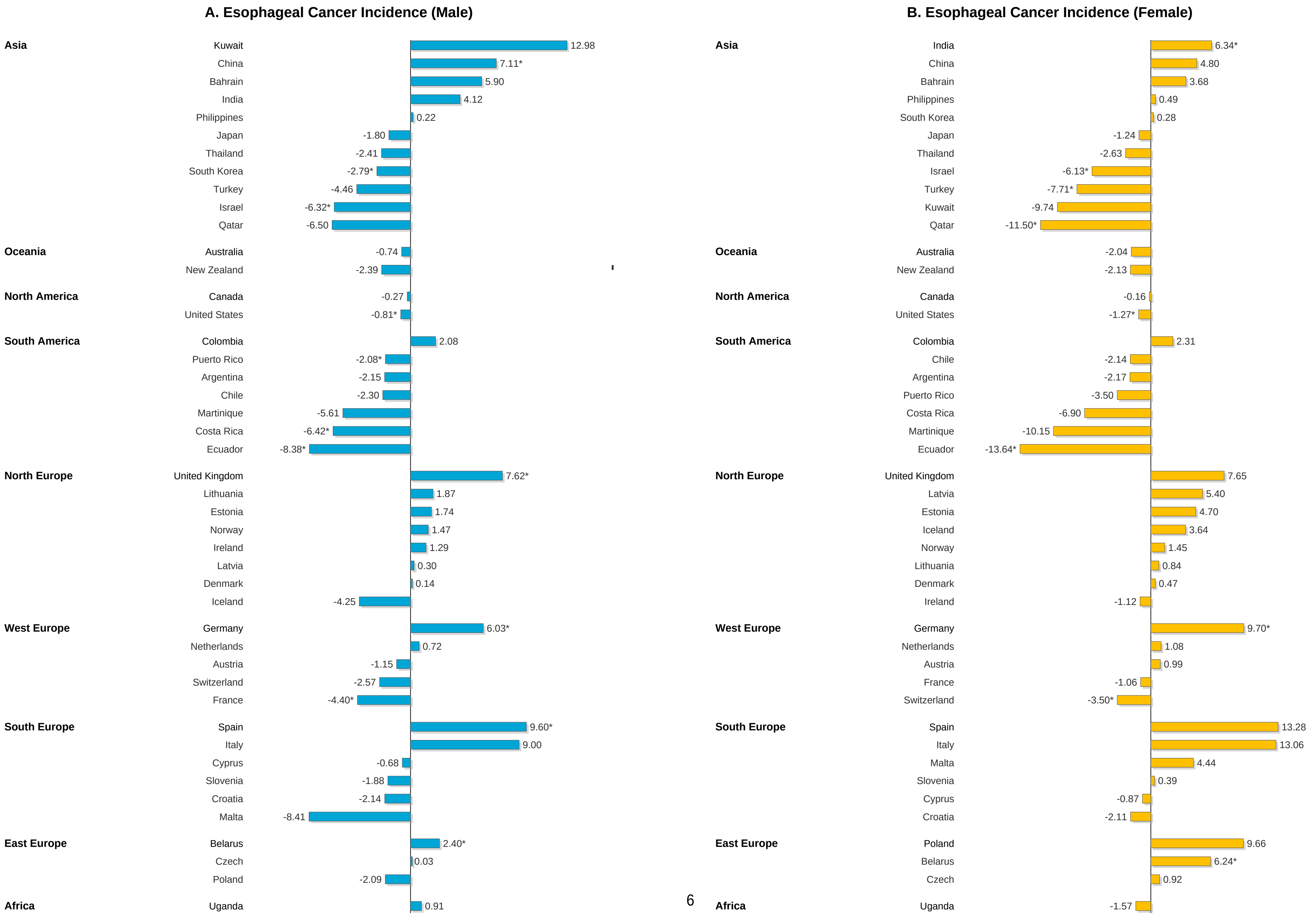

Supplementary Figure 4. Annual Average Percentage Change of esophageal cancer incidence by age.

From 2008 to 2017 by country (n=45). Two-sided Joinpoint regression test was used. Exact p values were provided in Supplementary Dataset 8, and statistically significant changes ($p < 0.05$) were marked with an asterisk (*).

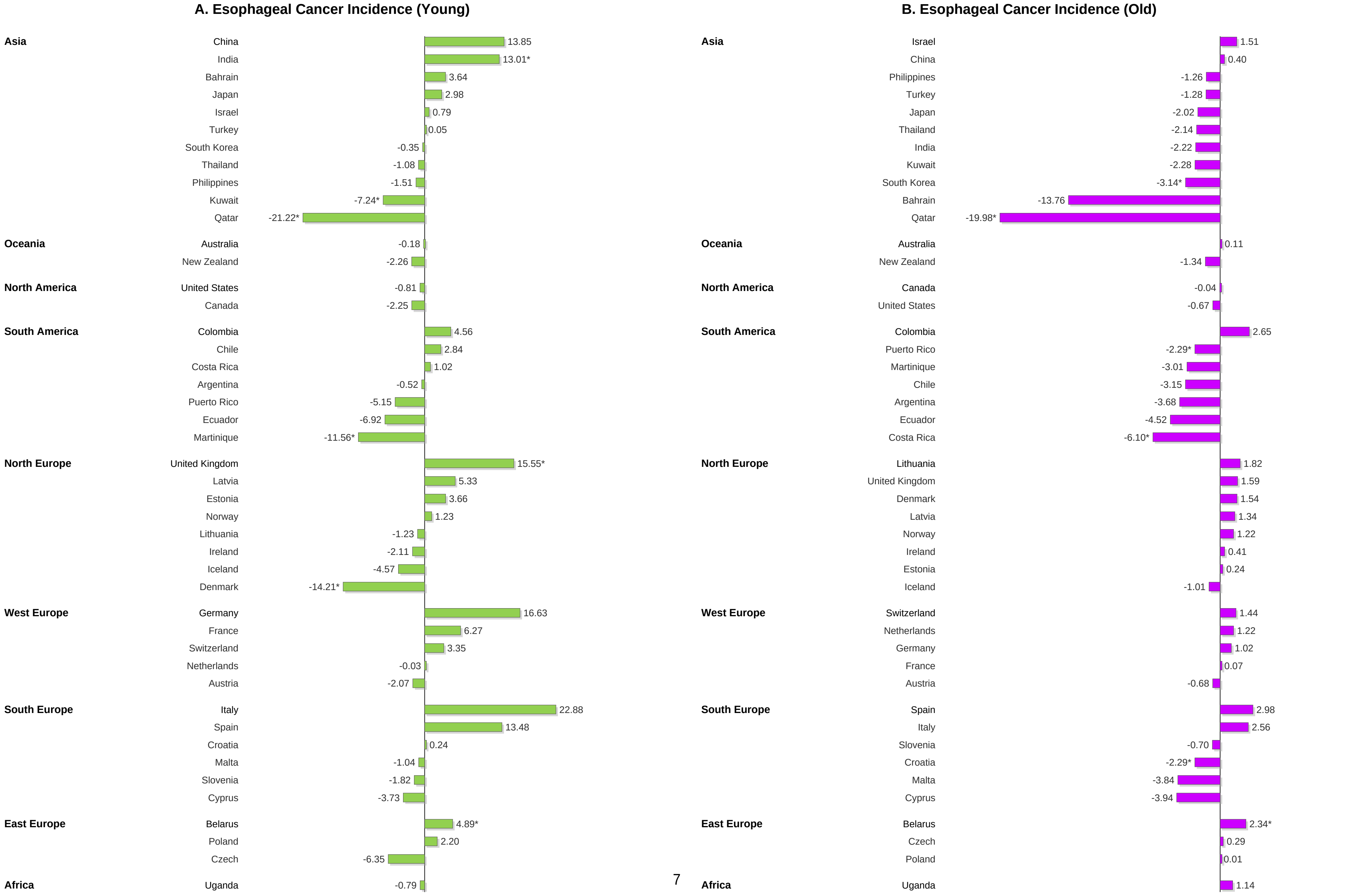

Supplementary Figure 5. Annual Average Percentage Change of ESCC incidence by sex.

From 2008 to 2017 by country (n=45). Two-sided Joinpoint regression test was used. Exact p values were provided in Supplementary Dataset 8, and statistically significant changes ($p < 0.05$) were marked with an asterisk (*).

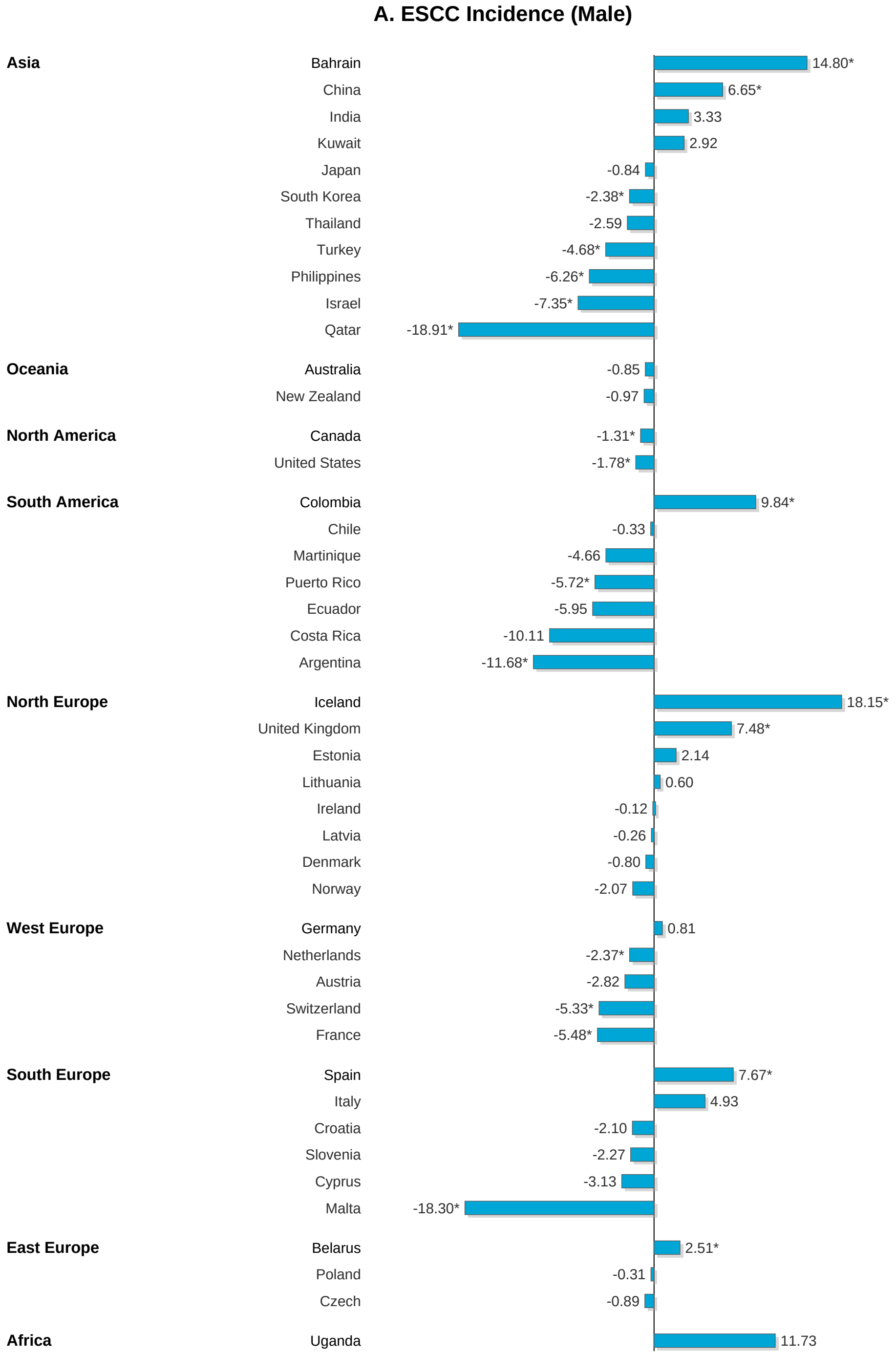

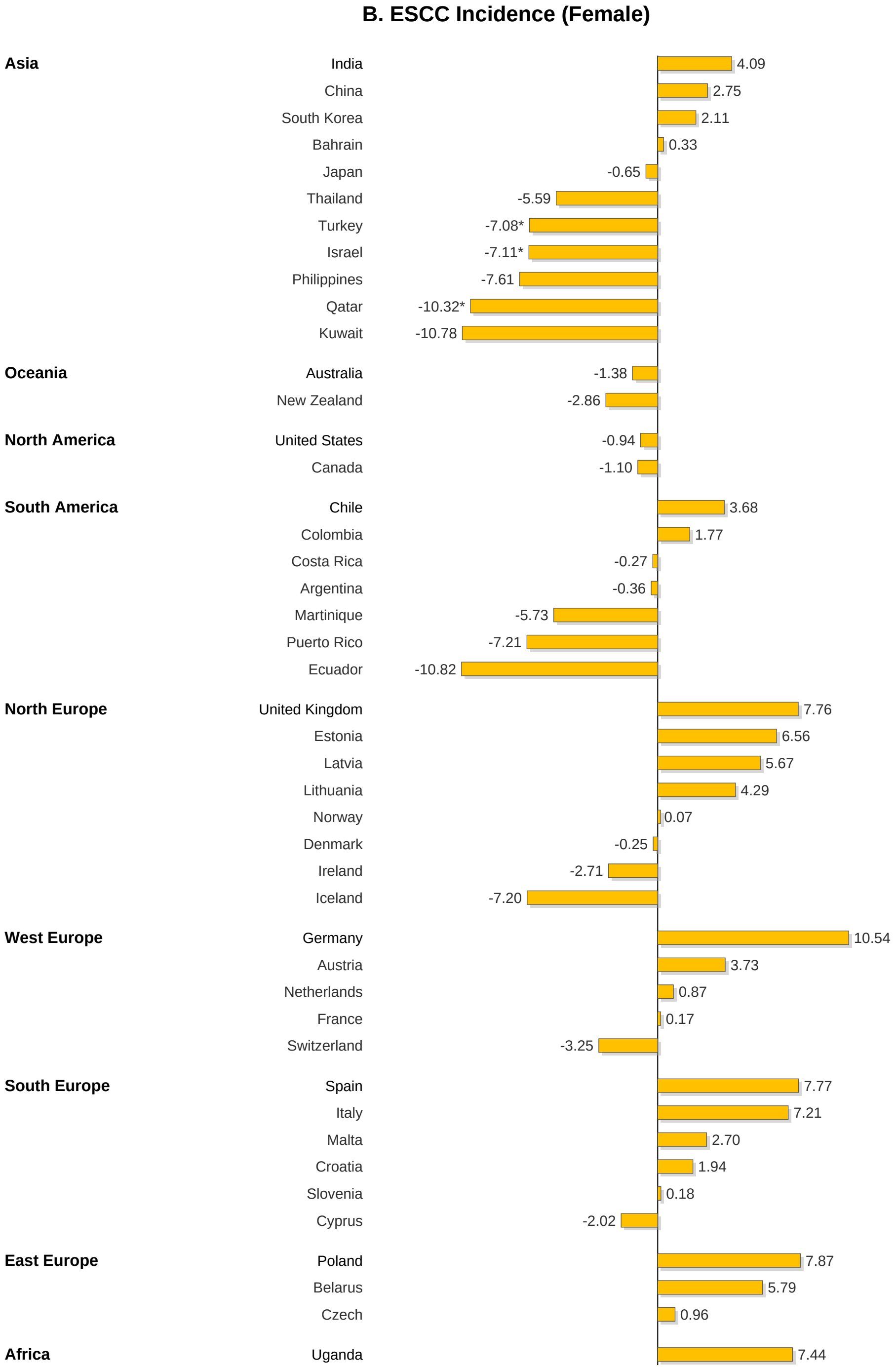

Supplementary Figure 6. Annual Average Percentage Change of ESCC incidence by age.

From 2008 to 2017 by country (n=45). Two-sided Joinpoint regression test was used. Exact p values were provided in Supplementary Dataset 8, and statistically significant changes ($p < 0.05$) were marked with an asterisk (*). Joinpoint regression could not be conducted for countries with zero incidence; therefore, their AAPC results are reported as NA.

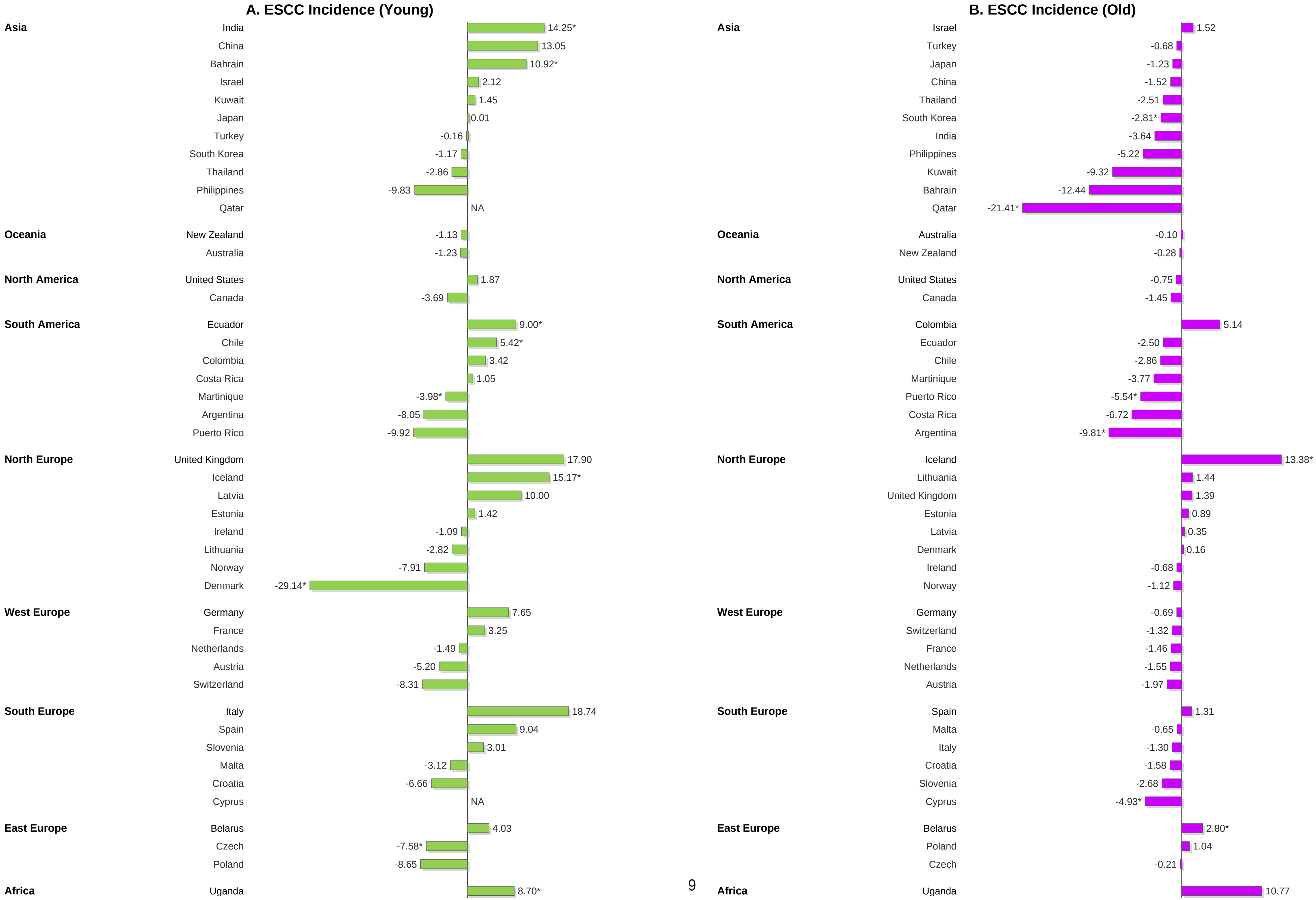

Supplementary Figure 7. Annual Average Percentage Change of EAC incidence by sex.

From 2008 to 2017 by country (n=45). Two-sided Joinpoint regression test was used. Exact p values were provided in Supplementary Dataset 8, and statistically significant changes ($p < 0.05$) were marked with an asterisk (*).

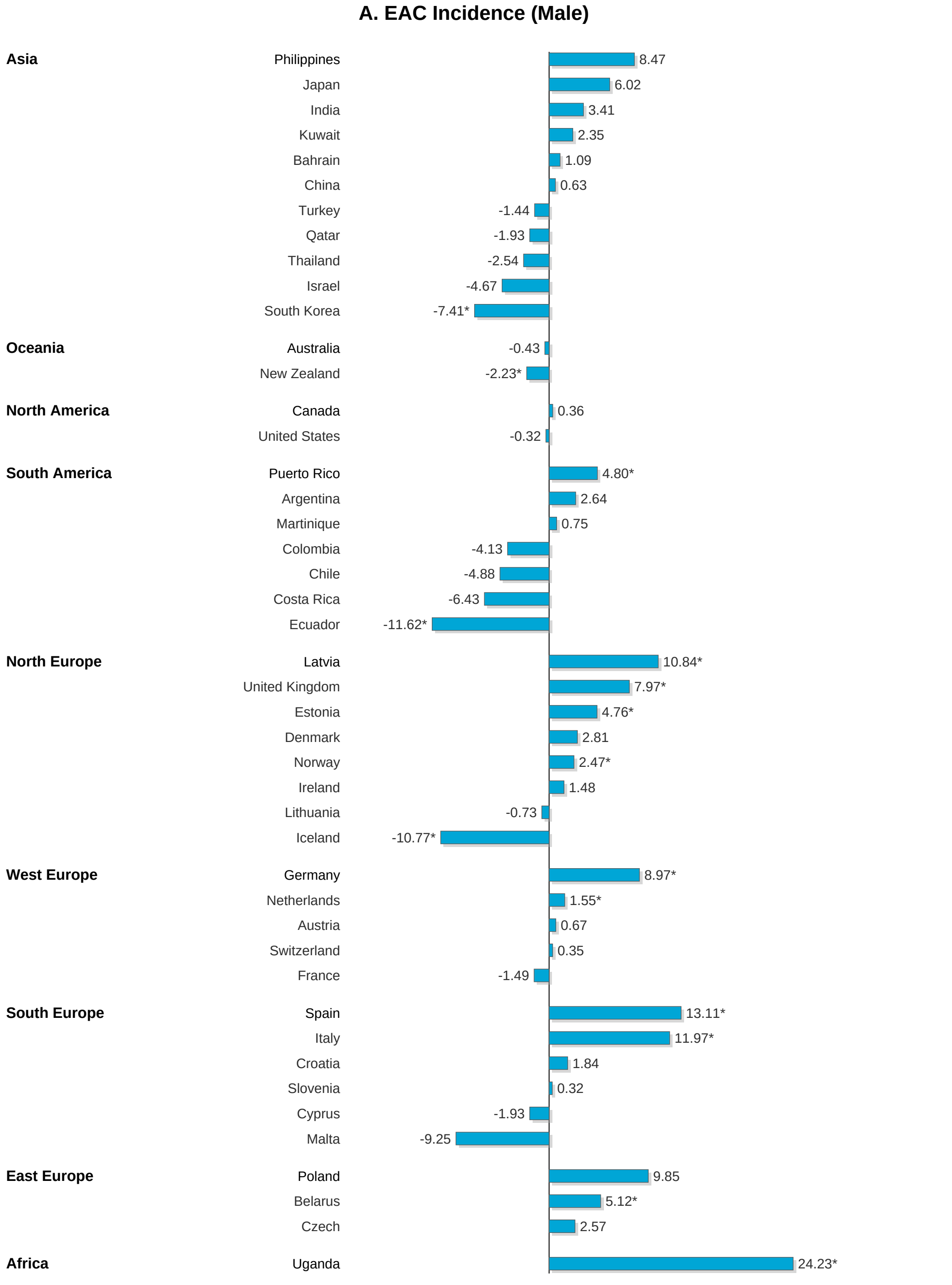

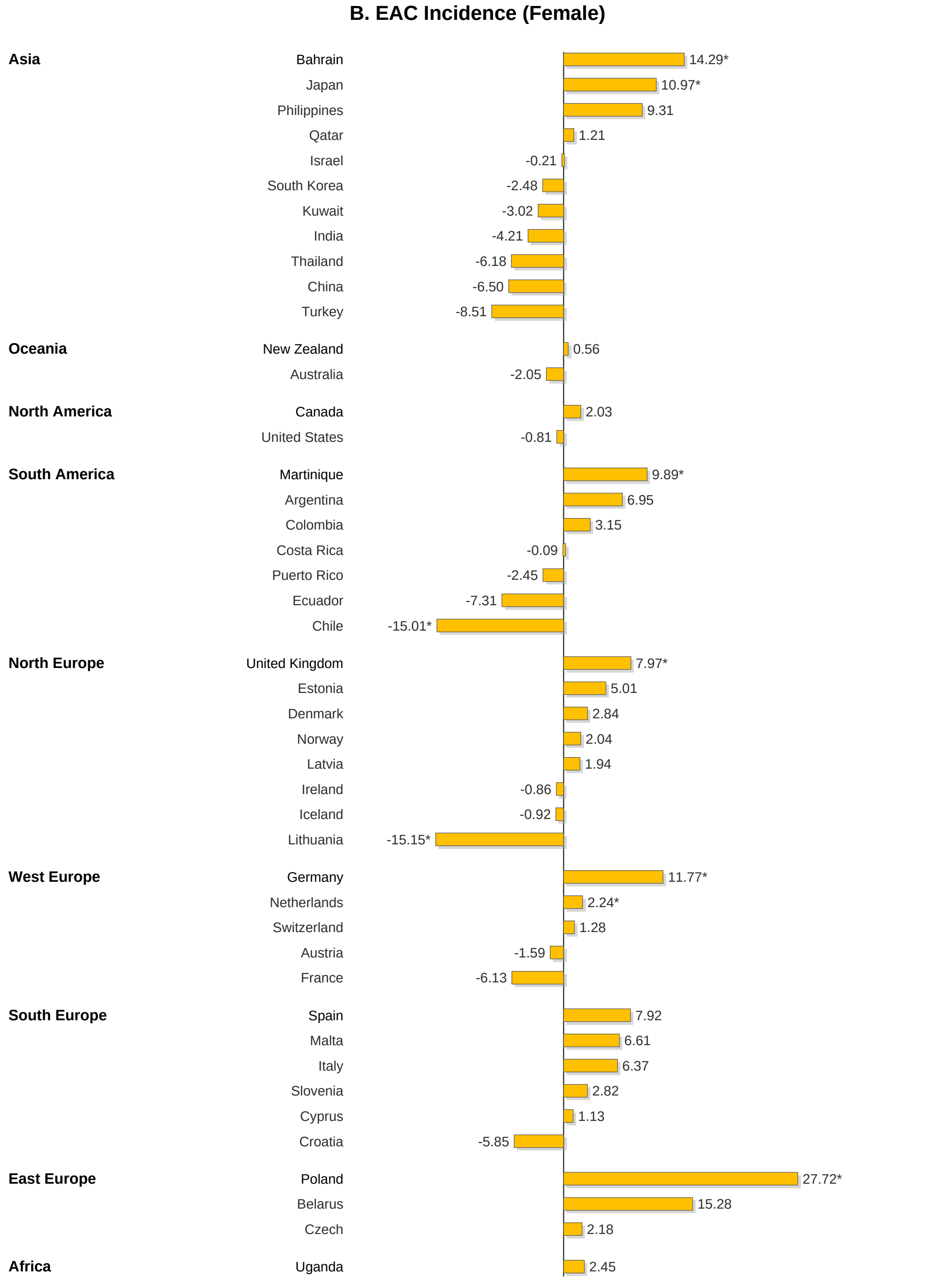

Supplementary Figure 8. Annual Average Percentage Change of EAC incidence by age.

From 2008 to 2017 by country (n=45). Two-sided Joinpoint regression test was used. Exact p values were provided in Supplementary Dataset 8, and statistically significant changes ($p < 0.05$) were marked with an asterisk (*).

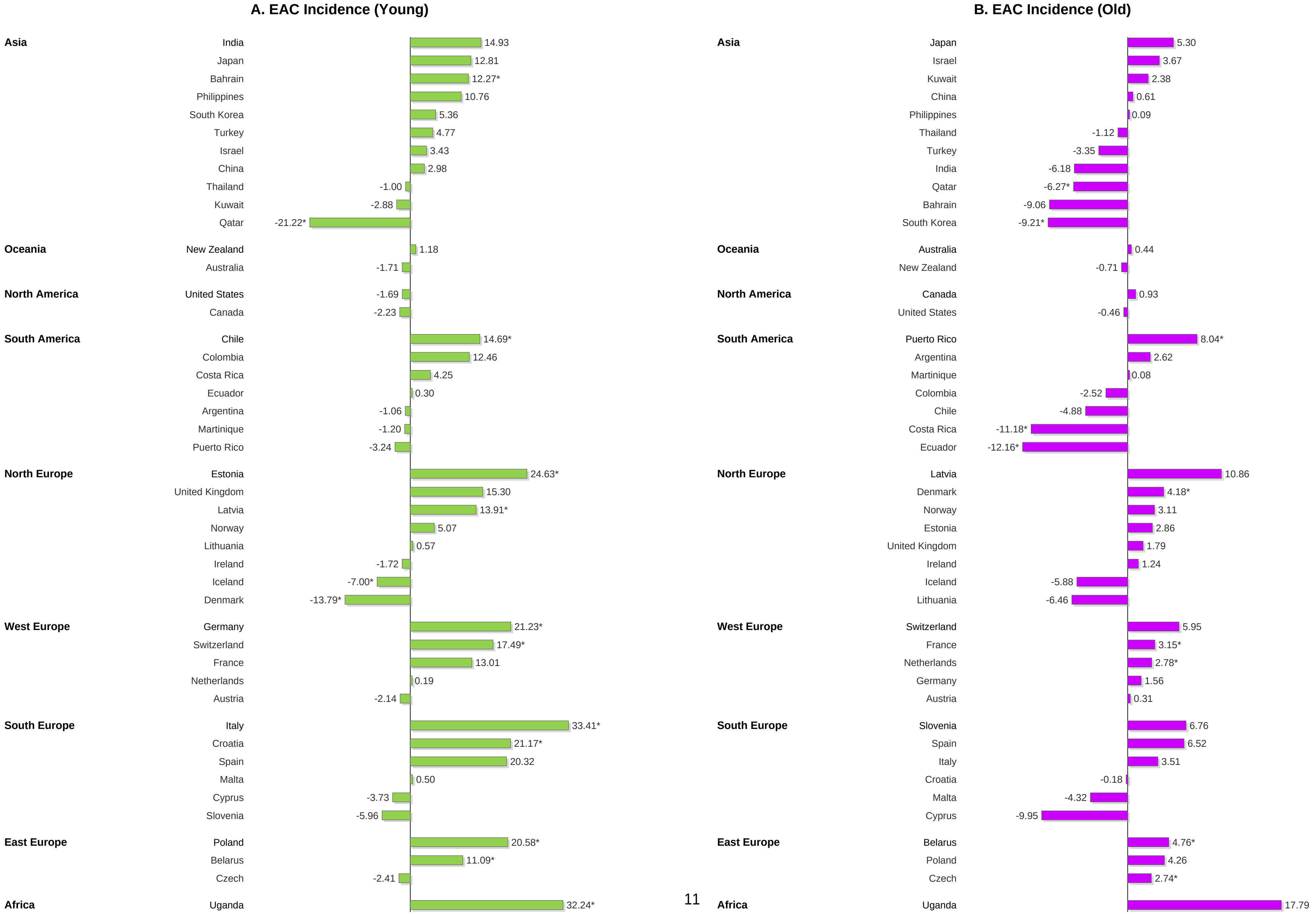

Supplement: Supplementary file 3 [file 41467_2026_76540_MOESM1_ESM.pdf]
