# Supplementary material for: Global incidence of esophageal cancer by histological and anatomical subtypes
Source: Nat Commun. 2026 Sep 1;17:10430. doi: 10.1038/s41467-026-76540-1 (PMC13631342; doi:10.1038/s41467-026-76540-1)
Supplement: Supplementary file 4 [file 41467_2026_76540_MOESM2_ESM.docx]

**Description of Additional Supplementary Files**

**Supplementary Dataset 1. Global incidence of esophageal cancer in 2022.**

This dataset reports esophageal cancer incidence estimates by region and country in 2022, including new cases and age-standardized rates for overall esophageal cancer, ESCC, EAC, upper- and middle-third esophageal cancer, and lower-third esophageal cancer. Estimates are descriptive; no statistical test was performed.

**Supplementary Dataset 2. Global incidence of esophageal cancer by sex, all ages, in 2022.**

This dataset reports esophageal cancer incidence estimates by sex and geography in 2022, including new cases and age-standardized rates for overall esophageal cancer, ESCC, EAC, upper- and middle-third esophageal cancer, and lower-third esophageal cancer. Estimates are descriptive; no statistical test was performed.

**Supplementary Dataset 3. Global incidence of esophageal cancer by age, both sexes, in 2022.**

This dataset reports esophageal cancer incidence estimates by age group and geography in 2022, including new cases and age-standardized rates for overall esophageal cancer, ESCC, EAC, upper- and middle-third esophageal cancer, and lower-third esophageal cancer. Estimates are descriptive; no statistical test was performed.

**Supplementary Dataset 4. Associations of risk factors with esophageal cancer.**

This dataset reports associations between risk factors in 2021 and esophageal cancer incidence. Associations were evaluated using two-sided linear regression models with log-transformed age-standardized incidence rate as the outcome (n=185). Results are shown as beta coefficients with 95% confidence intervals and exact p values; p values less than 0.05 are marked with an asterisk.

**Supplementary Dataset 5. Associations of risk factors with esophageal cancer (sensitivity analysis).**

This dataset reports associations between risk factors in 2012 and esophageal cancer incidence. Associations were evaluated using two-sided linear regression models with log-transformed age-standardized incidence rate as the outcome (n=185). Results are shown as beta coefficients with 95% confidence intervals and exact p values; p values less than 0.05 are marked with an asterisk.

**Supplementary Dataset 6. Associations of risk factors with esophageal cancer incidence by histological and anatomical subtypes.**

This dataset reports associations between risk factors in 2021 and esophageal cancer incidence by histological and anatomical subtype. Associations were evaluated using two-sided linear regression models with log-transformed age-standardized incidence rate as the outcome (n=185). Results are shown as beta coefficients with 95% confidence intervals and exact p values; p values less than 0.05 are marked with an asterisk.

**Supplementary Dataset 7. Associations of risk factors with esophageal cancer incidence by histological and anatomical subtypes (sensitivity analysis).**

This dataset reports associations between risk factors in 2012 and esophageal cancer incidence by histological and anatomical subtype. Associations were evaluated using two-sided linear regression models with log-transformed age-standardized incidence rate as the outcome (n=185). Results are shown as beta coefficients with 95% confidence intervals and exact p values; p values less than 0.05 are marked with an asterisk.

**Supplementary Dataset 8. Annual average percentage change (AAPC) of esophageal cancer incidence.**

This dataset reports Joinpoint regression results for overall incidence trends from 2008 to 2017 by country (n=45), summarized using annual average percentage change (AAPC) values. Trends were evaluated using two-sided Joinpoint regression tests, with AAPC estimates, confidence intervals, and exact p values reported; p values less than 0.05 are marked with an asterisk.

**Supplementary Dataset 9. Number of cancer registries by country covered in CI5 XII.**

This dataset provides the number of included cancer registries for each country, grouped by continent in CI5 XII. Registry counts are descriptive; no statistical test was performed.

**Supplementary Dataset 10. Joinpoint regression trend analysis of esophageal cancer incidence.**

This dataset reports Joinpoint regression results for segment-specific incidence trends from 2008 to 2017 by country (n=45), summarized using annual percentage change (APC) values. Trends were evaluated using two-sided Joinpoint regression tests, with APC estimates, confidence intervals, and exact p values reported; p values less than 0.05 are marked with an asterisk.
